# Differences in rhizosphere microbial communities between native and non‐native *Phragmites australis* may depend on stand density

**DOI:** 10.1002/ece3.6811

**Published:** 2020-09-29

**Authors:** Wesley A. Bickford, Donald R. Zak, Kurt P. Kowalski, Deborah E. Goldberg

**Affiliations:** ^1^ U.S. Geological Survey – Great Lakes Science Center Ann Arbor MI USA; ^2^ Department of Ecology and Evolutionary Biology University of Michigan Ann Arbor MI USA; ^3^ School for Environment and Sustainability University of Michigan Ann Arbor MI USA

**Keywords:** bacteria, fungi, oomycetes, rhizoplane, rhizosphere, soil conditioning

## Abstract

Microorganisms surrounding plant roots may benefit invasive species through enhanced mutualism or decreased antagonism, when compared to surrounding native species. We surveyed the rhizosphere soil microbiome of a prominent invasive plant, *Phragmites australis*, and its co‐occurring native subspecies for evidence of microbial drivers of invasiveness. If the rhizosphere microbial community is important in driving plant invasions, we hypothesized that non‐native *Phragmites* would cultivate a different microbiome from native *Phragmites*, containing fewer pathogens, more mutualists, or both. We surveyed populations of native and non‐native *Phragmites* across Michigan and Ohio USA, and we described rhizosphere microbial communities using culture‐independent next‐generation sequencing. We found little evidence that native and non‐native *Phragmites* cultivate distinct bacterial, fungal, or oomycete rhizosphere communities. Microbial community differences in our Michigan survey were not associated with plant lineage but were mainly driven by environmental factors, such as soil saturation and nutrient concentrations. Intensive sampling along transects consisting of dense monocultures of each lineage and mixed zones revealed bacterial community differences between lineages in dense monoculture, but not in mixture. We found no evidence of functional differences in the microbial communities surrounding each lineage. We extrapolate that the invasiveness of non‐native *Phragmites*, when compared to its native congener, does not result from the differential cultivation of beneficial or antagonistic rhizosphere microorganisms.

## Graphical abstract

We explored whether microbial communities differed in rhizosphere of native and non‐native *Phragmites autralis*. We found that rhizosphere communities were not different in most locations, but subtle differences could emerge in bacterial communities of dense patches. We concluded that rhizosphere microbial communities do not likely drive invasiveness of *Phragmites*, but may change as a consequence of ongoing invasions.

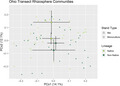

## 1 INTRODUCTION

The plant‐associated microbiome can dramatically influence plant performance and therefore may play a vital role in driving plant invasions in many ecosystems (Kowalski et al., 2015; Reinhart & Callaway, 2006). Plant‐microbial interactions span a spectrum from beneficial to antagonistic, and plants may perform better or worse than heterospecifics if their community of microorganisms functionally differs. If invasive plants associate with relatively fewer pathogens than native plants, they will realize performance advantages (Keane & Crawley, 2002; Reinhart & Callaway, 2006). Similarly, interaction with more mutualists may provide disproportionally stronger benefits to invaders, relative to native species (Reinhart & Callaway, 2006; Richardson et al., 2000).

Soil dwelling microorganisms may play a prominent role in a plant's invasiveness. For example, a recent meta‐analysis found that plant invasions can alter rhizosphere microbial communities, specifically increasing nitrogen mineralization, extracellular enzyme activity, and arbuscular mycorrhizal fungi (AMF) abundance, while decreasing soil pathogen and herbivore abundance (Zhang et al., 2019). Additionally, invasive plants may accumulate pathogens in the soil that are more virulent to native plants than themselves (Crocker et al., 2015; Mangla & Callaway, 2008). Consequently, a better understanding of plant‐microbial interactions and how they differ between native and invasive plant species will improve our collective understanding of the mechanisms underlying plant invasiveness and may ultimately improve invasive species management outcomes.

*Phragmites australis* is a cosmopolitan wetland grass with multiple lineages worldwide and is considered a model organism for studying plant invasions (Meyerson et al., 2016). Invasive to North America, the European lineage (*P. australis* haplotype M; hereafter, non‐native *Phragmites*) is highly productive and fast growing, often forming dense stands supporting a low overall species diversity. A native lineage in North America (*Phragmites australis* subsp. *americanus*, hereafter native *Phragmites*) is conversely characteristic of low nutrient, high‐diversity wetlands and is considered desirable for wildlife habitat (Price et al., 2013).

The microorganisms associated with *Phragmites* populations have been implicated in its performance in a variety of settings worldwide. For instance, in native European populations, bacterial and oomycete communities in the rhizosphere correlated with stages of decline in populations affected by reed die‐back syndrome (Bacci et al., 2018; Cerri et al., 2017). Likewise, several authors have suggested the key to understanding the invasive nature of non‐native *Phragmites* in North America may lie in microbial associations (Clay et al., 2016; Kowalski et al., 2015; Shearin et al., 2018). However, the sum of evidence for widespread differences in microbial assemblages between native and non‐native *Phragmites* lineages is mixed. For instance, Nelson and Karp (2013) found different rhizosphere pathogen communities (mainly *Pythium* spp.) associated with each lineage, although the total abundance of rhizosphere pathogens did not differ. They speculated that those differences may increase invasiveness of non‐native *Phragmites* due to enemy release (Keane & Crawley, 2002). Additional evidence indicates that differential virulence of pathogens might favor non‐native *Phragmites* over native *Phragmites* and especially over other native species (Crocker et al., 2015).

Importantly, Bowen et al. (2017) showed that bacterial communities in the rhizosphere differed dramatically among the dominant *Phragmites* lineages broadly distributed across the east and west coasts of North America. In fact, geographically distant *Phragmites* populations of the same lineage had more similar bacterial communities than neighboring populations of different lineages, suggesting that lineage‐specific cultivation drives rhizosphere community composition (Bowen et al., 2017). Despite this compelling evidence of lineage‐specific bacterial selection in the rhizosphere, the authors could not elucidate any functional link between bacterial communities and plant performance.

Several studies have found less support for differential microbial community cultivation between native and non‐native *Phragmites* lineages. For example, in tidal wetlands of the Chesapeake Bay region (Mid‐Atlantic coast of USA), *Phragmites* lineages cultivated dissimilar rhizosphere archaeal communities, but contrary to the findings of Bowen et al. (2017), rhizosphere bacterial communities did not differ between lineages (Yarwood et al., 2016). Likewise, our recent study examining root endophytes residing in native and non‐native *Phragmites australis* roots in the state of Michigan, USA, revealed that root bacterial, fungal, and oomycetes communities did not differ between native and non‐native *Phragmites* lineages (Bickford et al., 2018). Instead, root microbial communities were strongly influenced by environmental characteristics, such as soil saturation and nutrient status. Because microbial communities residing in native and non‐native *Phragmites* roots did not differ in either composition or function, there was no evidence to suggest that root endophytes contributed to the invasiveness of the non‐native lineage.

Plants may select for particular belowground microbial communities through release of specific root exudates or by altering the rhizosphere soil environment. For instance, in waterlogged soils, oxygen diffusion into the soil could select for more aerobic organisms in the root zone. Importantly, native and non‐native *Phragmites* differ vastly in their ability to aerate soils in the root zone, with the differences driven mostly by higher live stem density and a large number of senesced stems from previous years in invasive populations (Tulbure et al., 2012). Therefore, microbial community differences between *Phragmites* lineages may result from differences in soil oxygen concentrations and the strength of differences may depend on the stand age, density, and dominance of the patch.

Here, we expand upon our previous study on microbes internal to the roots of *Phragmites* (Bickford et al., 2018) to examine broad components of the rhizosphere soil microbiome (i.e., bacteria, fungi, and oomycetes) in native and non‐native *Phragmites* populations. Given the mixed evidence for distinction in rhizosphere microbial communities between *Phragmites* lineages, we sought to examine whether soil communities surrounding each lineage differed or, as with the root communities of the Great Lakes, were similar. Despite no differences found in roots (Bickford et al., 2018), rhizosphere communities may be driven by a separate set of factors such as differences in oxygen diffusion sensu Tulbure et al. (2012). Accordingly, stand density and dominance may play an important role in the strength of differentiation in microbial communities between lineages. This is the first study to explore rhizosphere soil microbes of multiple groups surrounding lineages of *Phragmites* in the Great Lakes region and is also the first to qualitatively address the impact of stand age, density, and dominance in rhizosphere community development.

We assessed the rhizosphere microbiome of each lineage to investigate the potential role of the rhizosphere microbiome in fostering the invasion of non‐native *Phragmites*. If performance differences between native and non‐native plant lineages are driven by their rhizosphere microbial communities, we would expect (a) the rhizosphere community of native and non‐native *Phragmites* to harbor compositionally dissimilar bacteria, fungi, and oomycete communities and (b) the non‐native lineage to associate with more mutualistic and/or fewer pathogenic microbes in rhizosphere soil.

We tested these hypotheses over a range of sites across Michigan, USA that varied in environmental conditions, thereby allowing us to explore additional drivers of microbial community composition, such as soil nutrient content and saturation. We further tested our hypotheses at two sites in the state of Ohio, USA, in which dense and extensive populations of native and non‐native *Phragmites* intergrade from nearly pure stands to mixtures of each. Intensive sampling along 20‐m transects at these two sites allowed us to explore (a) whether the degree of differentiation differed between dense monoculture stands and mixed plant community zones within the same environment and (b) whether differential rhizosphere cultivation between lineages was detectable at across spatial scales. We included multiple levels of soil proximity to host plant roots in paired samples (rhizoplane, rhizosphere, and bulk soil), allowing us to determine if either lineage cultivates a microbial community that is detectably different from the bulk soil community, and whether the strength or direction of cultivation differs by plant lineage.

## 2 MATERIALS AND METHODS

### 2.1 Site selection

Our study included 6 sites distributed across Michigan, USA with co‐occurring populations of native and non‐native *Phragmites* (hereafter Michigan Sites; Appendix S1: Table S1) and two sites in Ohio, USA, in which dense native and non‐native *Phragmites* stands co‐occur and mix (hereafter, Ohio Sites; Appendix S1: Table S1). Sampling protocols differed slightly between the two regions and are described in detail below.

### 2.2 Michigan sites

In August 2016, we sampled rhizosphere and bulk soils from native and non‐native *Phragmites* at 6 sites distributed across Michigan, USA (Appendix S1: Table S1). We selected sites that had at least 3 distinct patches of native and non‐native *Phragmites* in close proximity to one another, growing under similar environmental conditions (e.g., soil type, hydrology) with no recent history of invasive plant management (e.g., herbicide, burning). Due to the rarity of co‐occurring native and non‐native *Phragmites* populations that met these criteria (non‐native is rare and well‐managed in northern Michigan; native is rare in southern Michigan), patch size and density varied considerably among sites (1 m^2^ to 100 m^2^) and many patches were of low density. Exact stand age is unknown, but based on Great Lakes water level trends and historical aerial imagery, we can estimate that the stands in northern Michigan were <5 years old when sampled. The stands in southern Michigan appear <10 years old based on aerial imagery. We use the Ohio sites (described below) to assess whether patch size and density changes the extent of microbial community cultivation.

At each site, we morphologically identified all *Phragmites* patches as native or non‐native and leaf material from each was collected for later genetic confirmation of lineage using the methods of Saltonstall (2002). We classified the degree of soil saturation as either unsaturated, saturated, or saturated with standing water, and recorded depth of water (if over the surface) and the nature of surrounding vegetation. At each site, we collected rhizosphere and bulk soil samples in each of three native and three non‐native patches (one site did not have three distinct non‐native patches, see Appendix S1, Table S1). One ramet near the center of each patch was randomly selected for collection of paired rhizosphere and bulk soils. Using a serrated knife, we cut a 10‐cm diameter circle around the chosen ramet, exhuming subtending roots with adhering soil. The root ball was shaken to remove loosely associated soil. To sample rhizosphere soils, we then vigorously shook the root ball in a bag, saving the soil that fell off. Bulk soils were sampled outside of each *Phragmites* patch and paired with rhizosphere soils at the patch level. Leaf samples from the same stem were collected for tissue nutrient analysis. All samples were kept on ice until returned from the field.

### 2.3 Ohio sites

In September of 2017, we established two 20‐m transects within the Cedar Point National Wildlife Refuge, in Ohio USA (Appendix S1, Table S1). The transects were established where large, dense native and non‐native *Phragmites* co‐occur and intermix. Exact age of each transect location is unknown, but based on historical aerial imagery, stands appear to be >10 years old at the time of sampling. Each transect contained a high‐density zone of non‐native *Phragmites* dominance, a mixed zone containing both native and non‐native, and a high‐density zone of native *Phragmites* dominance. Each lineage's high‐density dominant zone was a near monoculture (i.e., included few other plant species at low abundance and did not include the opposite lineage); they will hereafter be referred to as monocultures. Samples were collected within 0.5 × 0.5 m plots at 2‐m intervals in the monoculture zones of both transects. In the mixed zones, samples were collected at 0.5‐m intervals in Transect 1 and 1.0 m intervals in Transect 2 (Transect 1 had 44 total sampling locations; 21 native, 23 non‐native; Transect 2 had 25 total sampling locations; 12 native, 13 non‐native. See Appendix S1, Table S1 for more details). Within each plot, we assessed plant species composition by counting the stems of each *Phragmites* lineage, identifying other plant species, and estimating total percent plant cover. One *Phragmites* ramet of each lineage was collected within each plot (1 sample in monoculture; 2 in mixed plots). Paired bulk and rhizosphere soils were collected as described above; bulk soils were collected adjacent to the plot in a zone of low stem density to avoid root influence. In addition, the entire root ball with adhering soil particles (rhizoplane soils) from the selected ramet was also collected and returned to the lab on ice.

### 2.4 Sample preparation

Samples collected from both sampling regions were prepared identically, except for the rhizoplane soils sampled from only the Ohio sites. For soil nutrient analysis, a subset of the bulk soil from each sample was passed through a 2‐mm sieve and oven dried at 60°C for 48 hr. Dried samples were ground with a mortar and pestle, and subsamples from each (0.5 g) were processed in duplicate in a Leco CNS2000 Analyzer (LECO®) to measure total carbon and nitrogen. Extractable soil phosphorus was determined colorimetrically following the Bray P1 extraction method (Bray & Kurtz, 1945).

Rhizosphere and the remainder of each bulk soil sample were passed through a sterilized 2‐mm sieve and stored at −80°C until DNA extraction. To obtain rhizoplane soils (Ohio sites), we collected ~10 coarse roots randomly from the root ball of each plant using sterile forceps. Sampled roots were placed into a sterile 50‐ml centrifuge tube with 30 ml of phosphate buffered saline (PBS). Tubes were vigorously shaken for 5 min, after which the roots were removed. Tubes were centrifuged at 8,000 *g* for 10 min. Supernatant was decanted, and the pellet was resuspended in 5–10 ml of supernatant in a 15‐ml tube and centrifuged again at 8,000 *g* for 10 min. After decanting supernatant, each tube containing pelletized rhizoplane soil was stored at −80°C until DNA extraction.

DNA was extracted from 50 mg (wet weight) of soils using Qiagen PowerSoil PowerLyzer DNA extraction kits. We used manufacturer protocols, with the exception of improvements to reduce ethanol contamination (e.g., extra spins, more frequent transfers to sterile tubes). DNA was eluted with molecular grade water. All genomic DNA extracts were verified by electrophoresis. Extracts were checked for quality on a NanoDrop UV/Vis spectrophotometer and concentration using a Quant‐iT PicoGreen dsDNA kit (Invitrogen).

All polymerase chain reactions (PCR) for each microbial group (i.e., fungi, bacteria, oomycetes) were performed using subsamples of the same template genomic DNA sample. Genomic DNA was diluted to ensure equimolar concentration of template DNA in each PCR reaction. Bacterial amplicons were generated using primers described in Kozich et al. (2013), which target the V4 region of the 16S rRNA gene. Fungal amplicons were produced using primers described by Taylor et al. (2016), which target the ITS2 region of the 5.8S rRNA gene. Oomycete amplicons were generated using primers adapted from Riit et al. (2016) and Taylor et al. (2016) that also target the ITS2 region of the 5.8S rRNA gene. See Appendix S1 Table S2 for specific primer sequences and PCR conditions. All PCR reactions were performed in triplicate using Phusion High Fidelity DNA Polymerase and master mix (New England BioLabs). Libraries were normalized using SequalPrep Normalization Plate Kit (Life technologies cat # A10510‐01) following the manufactures protocol for sequential elution. The concentration of the pooled samples was determined using Kapa Biosystems Library Quantification kit for Illumina platforms (Kapa Biosystems KK4824). The sizes of the amplicons in the library were determined using the Agilent Bioanalyzer High Sensitivity DNA analysis kit (cat# 5067‐4626). The final library consisted of equal molar amounts from each of the plates, normalized to the pooled plate at the lowest concentration. Amplicons were sequenced by the Microbial Systems Molecular Biology Laboratory (MSMBL) at the University of Michigan on the Illumina MiSeq platform, using a MiSeq Reagent Kit V2 500 cycles (Illumina cat# MS102‐2003), according to the manufacturer's instructions. Sequences were uploaded to the NCBI Sequence Read Archive under SRA accession number PRJNA601975.

### 2.5 Bioinformatic analysis

Raw bacterial sequence data were processed using mothur v1.39.5 (Schloss et al., 2009). Operational taxonomic units (OTUs) were clustered at 97% for bacterial sequences. Bacterial taxonomy was determined by comparing representative sequences to the taxa found in the SILVA database (Quast et al., 2018). Raw fungal and oomycete sequences were processed using QIIME2 (Bolyen et al., 2019) because QIIME2 can implement de novo sequence clustering of actual sequence variants (ASVs). Fungal sequences were clustered into OTUs at 97% similarity and assigned to taxonomy based on the UNITE database (Nilsson et al., 2013). Oomycete sequences were clustered at 97% similarity and assigned taxonomy in mothur using a custom oomycete‐specific database from the Barcode of Life Database (Ratnasingham & Hebert, 2007). Each microbial group was rarefied according to the sample that yielded the fewest number of sequences to ensure equal coverage across all samples. Bacteria were rarefied to 13,956 sequences, fungi to 11,036 sequences, and oomycetes to 1,000 sequences (Appendix S1, Figure S1). OTUs observed less than twice across all samples were removed from community analyses. Bacterial taxa were analyzed to identify the proportion belonging to common plant pathogen groups using genera found in Wood (1967) and Mansfield et al. (2012). To evaluate functional potential of fungal OTUs, we used FUNGuild (Nguyen et al., 2015), which parses fungal communities by trophic mode and functional guilds. We analyzed outputs at the trophic mode and guild level to group fungal taxa into putative functional groups. All oomycetes were assumed to be pathogens.

### 2.6 Statistical analyses

All statistical analyses were run in the R environment (R Core Team, 2018). We separately analyzed the data collected from the Michigan sites and Ohio sites because the sampling design differed between regions. We employed a multi‐stage approach in both regions to address whether the rhizosphere community of native and non‐native *Phragmites* harbored compositionally dissimilar bacteria, fungi, and oomycete communities. Permutational multivariate analysis of variance (PERMANOVA using the *adonis* function in the vegan package; Oksanen et al., 2019) tested whether plant lineage or site predicted significant microbial community differences among our samples. Homogeneity of Dispersions (PERMDISP using the *betadisper* function in the vegan package; Oksanen et al., 2019) further assessed whether microbial community samples differed in their degree of dispersion from their centroid. Finally, we used distance‐based redundancy analysis (db‐RDA using the *capscale* function in the vegan package; Oksanen et al., 2019) to constrain ordinations of Bray–Curtis distances by significant environmental drivers. Environmental drivers included in the model were determined by backward selection (using the *ordistep* function in the vegan package; Oksanen et al., 2019). To assess whether communities differed between rhizosphere and bulk soils at a given sampling location, we performed a partial db‐RDA of soil fraction effect on composition with sample as a conditioning variable. In addition, we explored microbial alpha diversity and relative sequence abundance at various taxonomic levels using only the rhizosphere data and differences with respect to site and plant lineage were assessed using two‐way ANOVA (Type III sum of squares using the *ANOVA* function in the car package; Fox & Weisberg, 2019). To further understand if either lineage showed evidence of microbial cultivation at increasing proximity to the root surface, we used paired *t* tests to explore differences in diversity between paired rhizosphere, rhizoplane (in Ohio sites only), and bulk soil samples. To understand the potential environmental drivers of site differences, we assessed the impact of soil nutrients and saturation on microbial diversity, including potential interactions with lineage using Analysis of Co‐Variance (ANCOVA using the *lm* and *ANOVA* functions).

In addition to the analyses mentioned above, we performed a few additional tests at the Ohio sites to take advantage of the unique sampling regime of the transects. To compare communities of bacteria between lineages in monoculture zones and in mixture zones, we used a pairwise PERMANOVA using a Bonferroni correction for multiple comparisons (Martinez Arbizu, 2018). We again used a partial db‐RDA to explore community differences between soil fractions and host lineages using sample plot as a conditioning variable to explore evidence of spatial structure in microbial communities.

For both regions, we calculated relative abundance of trophic modes determined by FUNGuild to assess the putative function of microbes and compare between lineages and used ANOVA to explore differences among sites and lineages and ANCOVA to determine impacts of soil nutrients and saturation on trophic mode relative abundance. All analyses used a threshold of *α* = 0.05 to assess significance, noting .05 < *p* < .1 as marginally significant. All R code, notes, and associated data can be accessed at https://doi.org/10.5066/P93BBZWU.

## 3 RESULTS

### 3.1 Michigan sites

**FIGURE 1:**
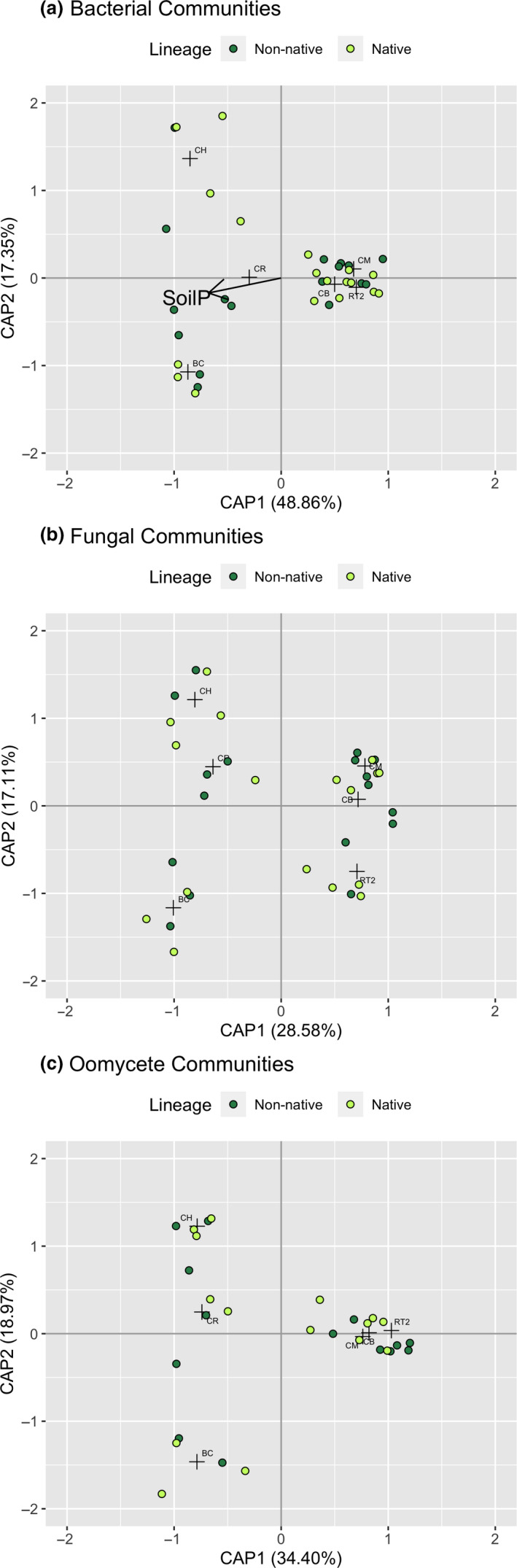
Distance‐based redundancy analysis plots of (a) bacterial, (b) fungal, and (c) oomycete communities found in the rhizosphere of the Michigan sites. Crosses indicate centroids of each site. Site was a significant predictor of variation in each microbial group. Vectors of significant environmental predictors also included (see Table 1 for statistics)

We found little evidence that native and non‐native plant lineages cultivated compositionally different microbial communities at the Michigan sites; communities of bacterial, fungi, and oomycetes did not significantly differ between *Phragmites* lineages (Table 1a, Figure 1). In contrast, sampling site was a significant predictor of variation in rhizosphere community composition for all three microbial groups (Table 1a). However, a significant test for homogeneity of multivariate dispersions (PERMDISP) suggested that the site differences in bacterial and oomycete communities were likely due to differences in dispersion around the centroids, rather than in mean composition (Table 1b). Soil phosphorus was important in structuring bacterial communities (Table 1, Figure 1).

**TABLE 1:** Results of (a) PERMANOVA analysis, (b) homogeneity of multivariate Dispersions (PERMDISP), and (c) distance‐based redundancy analysis (db‐RDA) for all three microbial groups in the rhizosphere

(a)	Site				Lineage				Site × Lineage			
	*df*	*F*	*R* ^2^	*p*	*df*	*F*	*R* ^2^	*p*	*df*	*F*	*R* ^2^	*p*
Bacteria	**5**	**3.605**	**0.379**	**.001**	1	1.084	0.023	.292	5	1.098	0.115	.273
Fungi	**5**	**2.191**	**0.284**	**.001**	1	1.033	0.027	.386	5	0.930	0.120	.791
Oomycetes	**5**	**1.769**	**0.275**	**.001**	1	1.097	0.034	.243	5	1.040	0.162	.314

(b)	Site			Lineage			
	*df*	*F*	*p*	*df*	*F*	*p*
Bacteria	**5**	**3.553**	**.014**	1	0.497	.520
Fungi	5	0.579	.715	1	0.095	.760
Oomycetes	**5**	**2.710**	**.046**	1	0.678	.431

(c)	Site			Soil P			
	*df*	*F*	*p*	*df*	*F*	*p*
Bacteria	**5**	**3.617**	**.001**	**1**	**1.671**	**.038**
Fungi	**5**	**2.216**	**.001**	(not in best model)		
Oomycetes	**5**	**1.746**	**.001**	(not in best model)		

Note

For PERMANOVA and PERMDISP, model included only Site and Lineage; the db‐RDA model used backwards selection to select the most significant variables for the model. Note that Lineage was not selected in the best model in the db‐RDA for any microbial group. Bold values indicate significance at α = 0.05.

**FIGURE 2:**
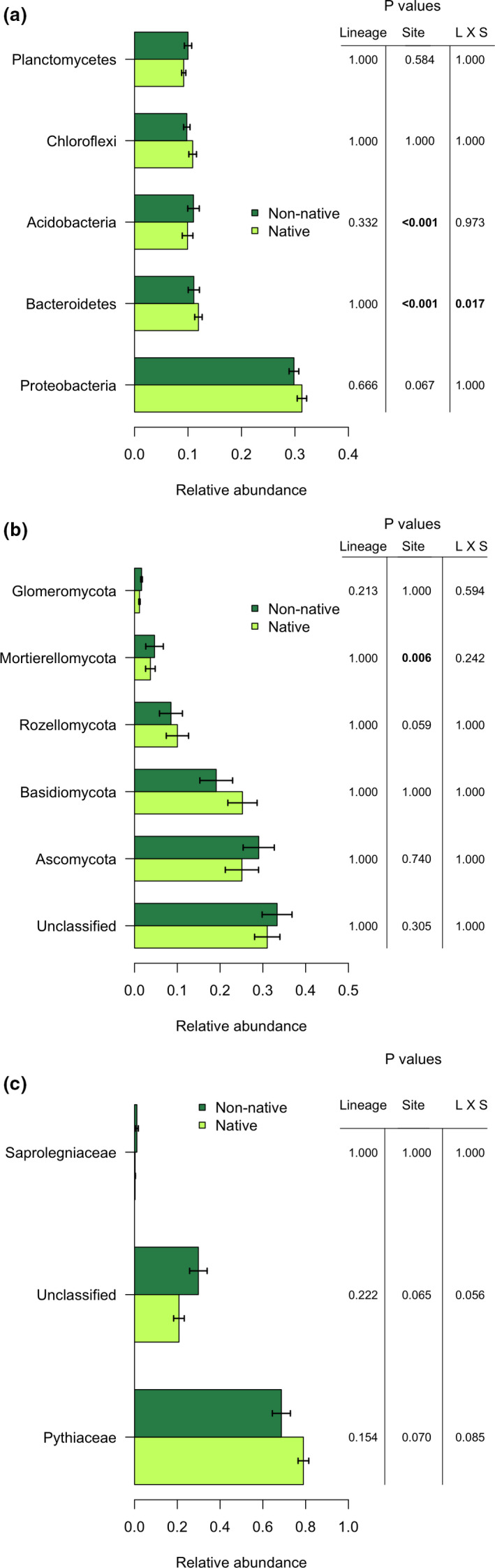
Relative abundance of dominant phyla of (a) bacterial, (b) fungal, and (c) oomycete families found in the rhizosphere. *p* values from a two‐way ANOVA with Type III sum of squares. Bonferroni correction applied for multiple comparisons. Significant *p* values in bold

Relative abundance of particular microbial phyla found in the rhizosphere also did not strongly differ by plant lineage, providing further evidence that native and non‐native plant lineages do not cultivate distinct microbial communities. Many of the most abundant bacterial phyla were differentiated among sites (Figure 2a), mainly driven by saturation (Appendix S1, Figure S2, Table S3). Soil saturation was a major factor in these differences among sites as it significantly affected abundance of most bacterial phyla (Appendix S1, Figure S2, Table S3). Proteobacteria (*r* ^2^ = .218, *p* = .002) and Chloroflexi (*r* ^2^ = .143, *p* = .014) increased with degree of saturation whereas Acidobacteria decreased (*r* ^2^ = .525, *p* < .001).

Abundance of fungal phyla also did not differ between native and non‐native *Phragmites* rhizosphere soil, although some common phyla differed among sites (Figure 2b). Soil saturation was a significant determinant of Mortierellomycota abundance (ANCOVA *p* < .001), such that it decreased in saturated soil (*r* ^2^ = .404, *p* < .001; Appendix S1, Figure S3, Table S4). The majority of oomycete sequences recovered belonged to Pythiaceae. Site and lineage interacted marginally in affecting Pythiaceae abundance (*p* = .076) as well as abundance of unclassified oomycetes (*p* = .051). Soil saturation and plant host lineage significantly interacted in affecting Pythiaceae (ANCOVA *p* = .018) and unclassified oomycete relative abundance (ANCOVA *p* = .021) such that the non‐native lineage hosted slightly less Pythiaceae (*r* ^2^ = .398, *p* = .016) and more unclassified oomycetes (*r* ^2^ = .377, *p* = .011) in saturated sites (Appendix S1, Figure S4, Table S5). The phylogenetic resolution of our recovered sequences did not allow us to compare abundance of Pythiaceae genera or species between *Phragmites* lineages.

We examined the differences in community composition and diversity between rhizosphere and bulk soil samples across the Michigan sites to provide additional context to the lack of community differences seen in rhizosphere communities between the lineages. On average, microbial community composition did not differ between bulk and rhizosphere soils for all three microbial groups (Appendix S1, Figure S5), and these results hold for both lineages. Results were similar when spatial structure was accounted for by pairing at the patch level: communities of bacteria, fungi, and oomycetes still did not differ in composition between the bulk and rhizosphere soils (Table 2). Diversity of bacteria, fungi, and oomycetes also did not differ between rhizosphere and bulk soil samples, when compared between pairs of co‐collected samples (Table 3; Appendix S1, Figure S6).

**TABLE 2:** Partial distance‐based redundancy analysis (db‐RDA) statistics comparing community composition of paired bulk and rhizosphere soils in the Michigan sites

	Sum of squares	*F*	*p*
Bacteria	0.132	1.08	.358
Fungi	0.288	1.28	.109
Oomycetes	0.313	1.03	.428

Note

Sample pair was defined as a conditioning variable to remove variation associated with sample location.

**TABLE 3:** Paired *t* test statistics comparing inverse Simpson diversity of paired bulk and rhizosphere soils

	Paired ‐ *t*	*df*	*p*
Bacteria	0.414	31	.682
Fungi	−1.101	31	.280
Oomycetes	0.479	25	.636

Note

Separate paired *t* tests within lineage were also nonsignificant.

### 3.2 Ohio sites

**FIGURE 3:**
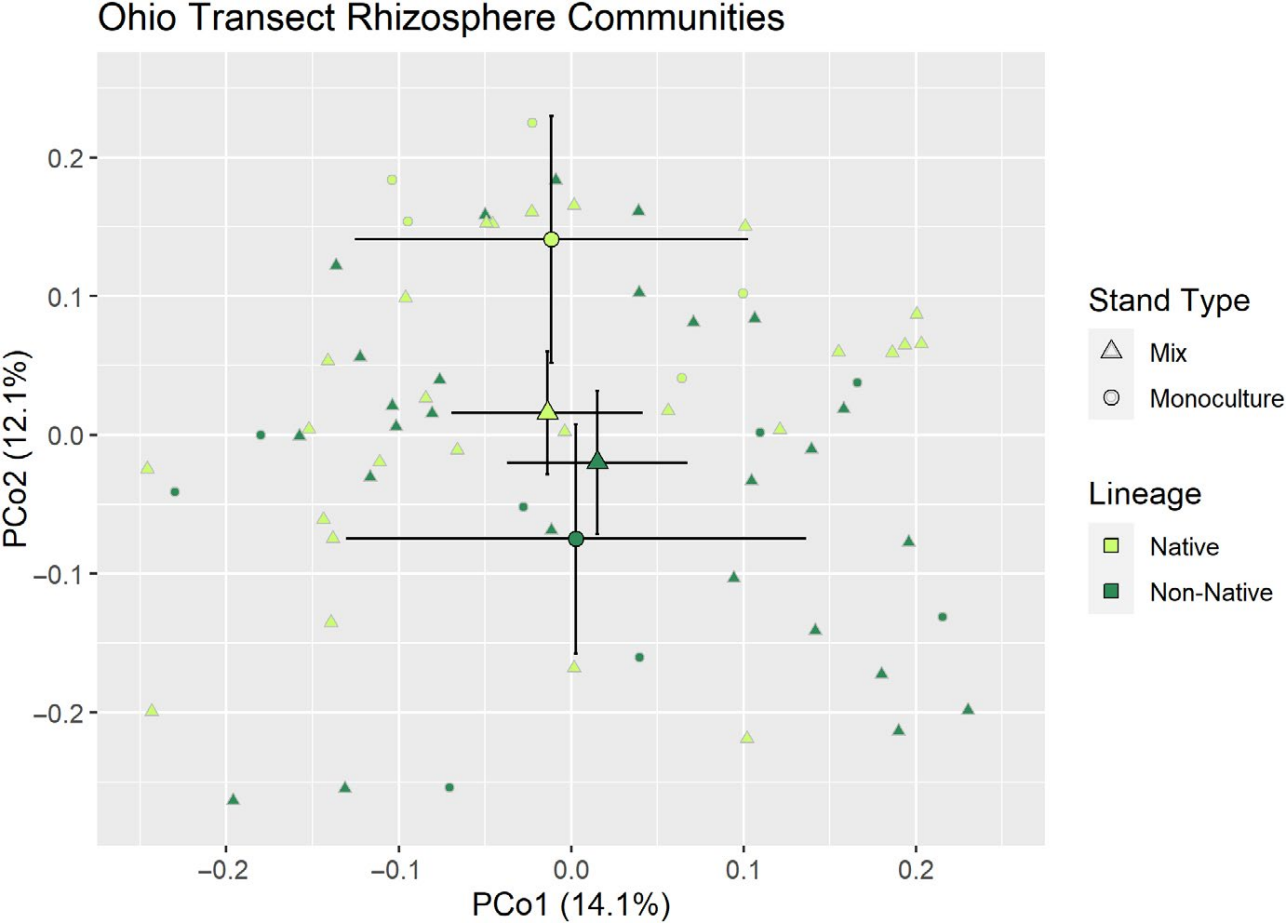
Principle coordinate analysis of Bray–Curtis distances between rhizosphere bacterial communities in the Ohio sites. Large points indicate centroids of each group with error bars denoting 95% confidence intervals

The intensive sampling arrangement at the Ohio sites allowed us to explore bacterial cultivation at a finer scale than we were able at the Michigan sites and illuminated some subtle, but important bacterial community differences between lineages. First, we compared the rhizosphere bacterial communities between lineages in both the monoculture and mixed zones. This analysis illustrated that lineage effects on rhizosphere bacterial communities depend on the relative density of natives and nonnatives (lineage by stand type interaction; PERMANOVA *r* ^2^ = .023, *p* = .070). Specifically, rhizosphere communities differed between monocultures of the two lineages (Figure 3; Pairwise PERMANOVA *r* ^2^ = .159, *p* = .048, Pairwise PERMDISP *p* = .826) while the lineages did not differ in mixtures (Figure 3, Pairwise PERMANOVA, *p* = 1.000, Pairwise PERMDISP *p* = .852, Appendix S1, Table S6).

We also explored the rhizoplane soils for evidence of differential cultivation between lineages. Lineage was a marginally significant predictor of variation across all plots (PERMANOVA *p = *.075); however, it only accounted for ~2% of the variation in community composition and the differences may be caused by differential dispersion between the groups (PERMDISP *p = *.023). Rhizoplane communities of different lineages, therefore, show little separation graphically (Appendix S1, Figure S7). Thus, across mixed and monoculture zones, we found no evidence of differentiation in microbial communities between lineages, even at a tight proximity to root. Interestingly, differences in rhizoplane soils by lineage did not seem to depend on stand dominance or density as pairwise comparisons showed no differences in community between lineages in monoculture stands (*p* = 1.000, Appendix S1, Table S7), and however, this result may have been influenced by the small sample sizes of rhizoplane soils in monocultures (*n* = 8 in non‐native, *n* = 3 in native, Appendix S1, Table S1).

**FIGURE 4:**
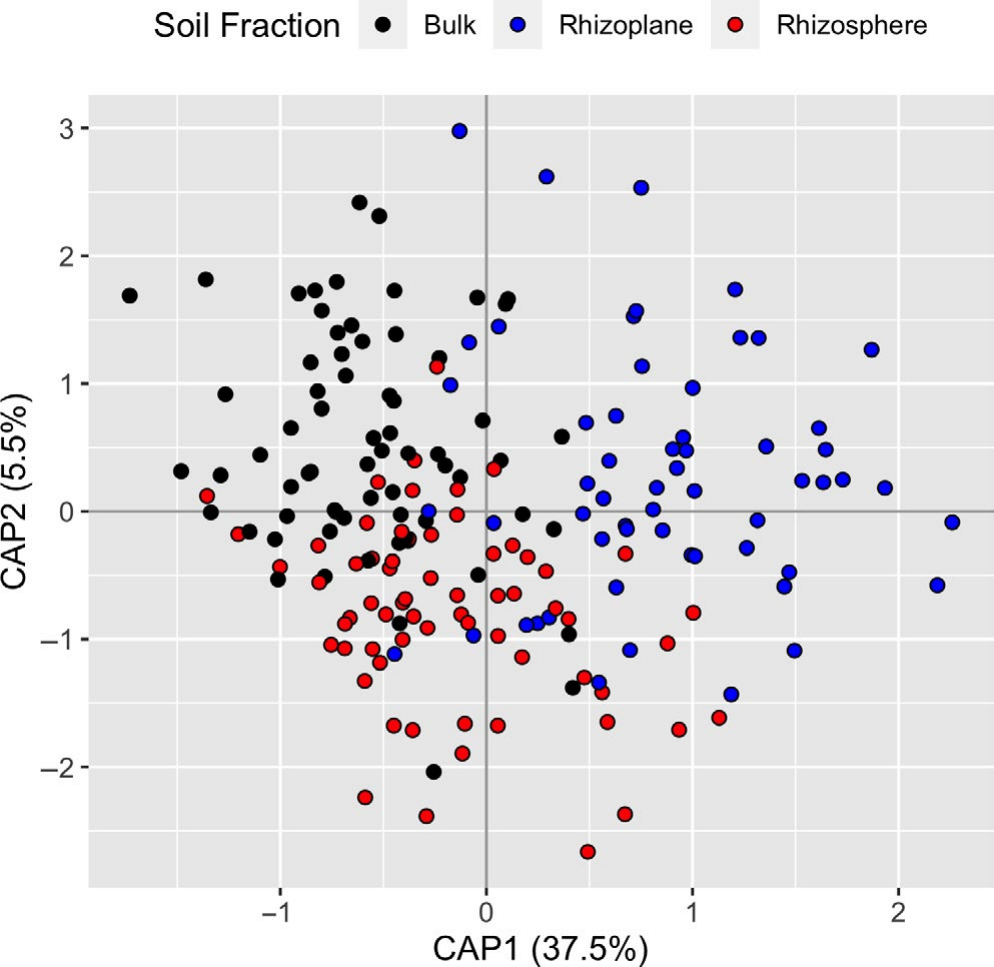
Comparison of bulk, rhizosphere, and rhizoplane soil bacterial communities paired at each plot along the Ohio transects. When sample location is set as a conditioning variable in a constrained ordination (db‐RDA), communities clearly separate depending on their proximity to the root surface. Soil fraction is a significant predictor of variation at the plot level (*F* = 8.8593, *p* = .001) but lineage is not (*F* = 1.0239, *p* = .338)

Next, to determine the extent to which *Phragmites* lineages were cultivating microbes near the root surface, we took advantage of the paired soil sampling design and compared the bacterial composition and diversity in the rhizoplane to both rhizosphere and bulk soil. There was evidence of some spatial structure in soil fractions as communities of bacteria differed significantly between bulk, rhizosphere, and rhizoplane soils paired at the plot level (partial db‐RDA, sum of squares = 1.7973, *F* = 8.8593, *p* = .001, Figure 4). In addition, rhizosphere soils were more diverse then the adjacent paired bulk soil and more diverse than paired rhizoplane. Rhizoplane was not different in diversity from bulk soil (Appendix S1, Figure S8). This suggests that more microbial species are present in the more “biologically active” zone of the rhizosphere compared to bulk soil, but only a subset of those are present in the still more narrowly defined zone of the rhizoplane.

### 3.3 Functional determination

**FIGURE 5:**
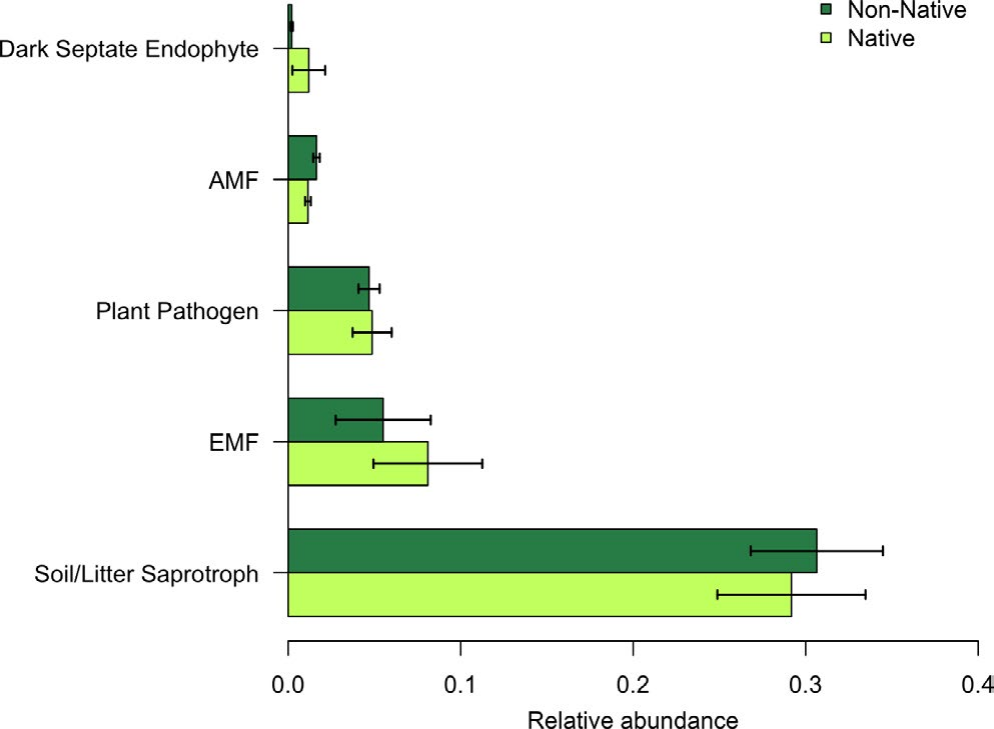
Relative abundance of dominant fungal functional groups found in the rhizosphere. Results of a two‐way ANOVA with Type III sum of squares verified that no comparisons between sites, lineages, or their interactions were significant at *α* = 0.05

Putative functional determinations of the microbial taxa in the rhizosphere revealed little to explain mechanisms of invasion. Only 0.5% of the bacterial sequences recovered belonged to known bacterial plant pathogens in the Michigan sites, and of that small portion, potential bacterial pathogens were not differentially abundant between native and non‐native lineages (ANOVA *F* = 1.575, *p = *.215). Potential pathogens made up 1% of the bacterial sequences in the Ohio sites and also did not differ in abundance between lineages (ANOVA *F* = 0.119, *p = *.731). Fungal functional determinations produced a similar result. First, 32.5% of all fungal sequences could not be classified at even the phylum level, leaving their functional potential also unknown. Of the classified sequences that matched the FUNGuild database, the majority were likely soil or litter saprotrophs. While small portion (~3%) were known plant pathogens, the proportion recovered from native rhizospheres was not different from non‐native (Figure 5). Likewise, none of the other functional groups, including the group that makes up the most common fungal mutualists, arbuscular mycorrhizal fungi, differed in relative abundance among sites or between plant lineages. We assume that all oomycete groups are pathogenic and although the relative abundance of one dominant family of pathogens, Pythiaceae, was marginally greater in the native lineage, the relative abundance of unclassified oomycetes (likely matching uncultured oomycetes) differed in the opposite direction (Figure 2c). Given the lack of consistency in lineage differences between oomycete families, we do not have compelling evidence that native *Phragmites* receives higher oomycete pathogen pressure than non‐native.

## 4 DISCUSSION

We found negligible evidence that native and non‐native *Phragmites* cultivated microbial communities that drive the differences in invasiveness that exist between them. Communities cultivated by each plant lineage were not different for any microbial group we examined at the Michigan sites, and we could find no meaningful differences in functional potential across all Michigan sites. The strongest evidence for differential cultivation comes from the Ohio sites in which native and non‐native monocultures, but not mixtures, significantly differed in their rhizosphere bacterial communities. The monoculture zones of the Ohio sites in which much of the difference occurred were more dominant, denser, and likely older than the sampling locations in Michigan. We argue below that the observation of differences only in the more dominant, denser patches suggests that rhizosphere microbial community differences are likely a consequence of invasion rather than a cause.

Our results contrast with two previous studies. Nelson and Karp (2013) explored rhizosphere pathogens and found that oomycete communities differed between native and non‐native *Phragmites* populations in New York, USA. Although our study did find marginal site × lineage interactions in relative abundance of Pythiaceae and unclassified Oomycetes, we did not find overall differences in community composition. It is possible this disparity arises due to sequencing depth in these respective studies. Nelson and Karp (2013) used a different sequencing platform that allowed much longer reads than our study (~475 bp vs. ~275 bp). The shorter reads and lower phylogenetic resolution in our study may have contributed to the smaller breadth of oomycete families we observed, thereby affecting community composition. However, in a study of endophytic root communities in the same Michigan sites as studied here, Bickford et al. (2018) found no difference in oomycete communities between *Phragmites* lineages using the same phylogenetic resolution as the rhizosphere data from Nelson and Karp (2013). Therefore, our results may accurately reflect the oomycete communities, but the lack of differences observed between *Phragmites* lineages in these two Great Lakes studies could plausibly be a reflection of the small, low density Michigan patches sampled.

Our results also contrast with those found in rhizosphere bacterial communities by Bowen et al. (2017). These investigators reported that *Phragmites* lineages cultivated consistent and distinct bacterial communities in the rhizosphere, regardless of geography, environmental characteristics, or temporal variation. The lack of consistency between our studies is surprising, and there are no clear ecological explanations that resolve the differences. For instance, while their dataset includes samples collected from *Phragmites* populations along the east, west, and Gulf coasts of the United States, their sites span a broad range of tidal influence and salinity regimes. Therefore, differences in salinity, hydrology, or both between our studies are not likely responsible for the different patterns observed. Instead, we argue that stand density and degree of dominance may explain the contrast in results.

Bowen et al. (2017) focused primarily on well‐established, large, dense *Phragmites* stands in which density differences between lineages may have been prominent. In contrast, our Michigan sites were comprised of smaller stands of each *Phragmites* lineage, due to the lack of sites with large, dense patches of both lineages. One potential consequence of differences in density is soil oxygen concentration. Non‐native *Phragmites* has a much higher ventilation efficiency than native *Phragmites*, thereby leading to a more oxygenated rhizosphere; this effect is thought to arise from a higher density of *Phragmites* stems in non‐native stands (Tulbure et al., 2012). In anoxic wetland soils, an increase in the soil oxygen concentration could plausibly change the composition of bacterial communities, such that more aerobic microbes are present. We speculate that the lack of differences observed in our sites could arise from the small, less dense patches sampled and correspondingly small differences in ventilation between native and non‐native lineages at our sites. The fact that the only place where we found differences between lineages was in the dense monoculture zones of the Ohio sites is consistent with this potential mechanism and indicates that dominance may factor into the degree of bacterial community differentiation between lineages, wherein high density, dominant patches may be more likely to host different bacterial communities. Future work should explicitly explore the effects of stand size, density, and soil oxygen concentrations on differential rhizosphere cultivation.

Data from our Michigan sites suggest the patterns in the rhizosphere microbiome largely mirror those of the root microbiome, where we also found no differences between *Phragmites* lineages across three major microbial groups (Bickford et al., 2018). In exploration of the root microbiome, Bickford et al. (2018) speculated that roots may select similar microbial inhabitants across lineages, despite the differences that may occur in the rhizosphere. However, the data presented here suggest, at least in low density patches, the rhizosphere microbial communities are driven by the environment, as they seem to be in the roots. Another recently published study of root endophyte communities focused on well‐established, high‐density native and non‐native *Phragmites* stands and found significant community differences between lineages (Gonzalez Mateu et al., 2020). Therefore, root community differences may also be related to stand density and dominance.

The cumulative evidence from our studies of roots, rhizosphere, and bulk soil suggest that at low densities, *Phragmites* lineages do not affect microbial communities differently, but as high‐density monocultures establish, dissimilarity in bacterial communities emerges. The functional implication of this dissimilarity is unresolved in regard to invasive capacity. In low density patches, environmental characteristics such as water saturation and soil nutrient content, but not lineage were strong determinants of community composition both in the roots (Bickford et al., 2018) and in the rhizosphere (this study). We speculate that the differences at high density are a consequence of a successful invasion, rather than driving differential success at the initial stages of invasion. We cannot separate the effects of stand age from stand density and dominance as *Phragmites* patches become denser and more dominant with time. Therefore, it is possible that as stands mature, becoming denser and more monotypic, dissimilarities in the belowground microenvironments of dense patches of different lineages may drive differences in belowground microbial communities. Subtle microbial community differences could potentially enhance invasiveness if they increased the interaction with mutualistic microbes or conditioned soils to the detriment of other native plants. However, direct comparisons of changes in microbial community function with stand age were outside of the scope of this study.

Our evidence also suggests that in small, less dense stands, neither *Phragmites* lineage cultivates a community that is substantially different from the surrounding bulk soil. We could not distinguish the communities of any microbial group between bulk and rhizosphere soils at the Michigan sites. Conversely, when we looked for evidence of cultivation at a fine spatial scale at the Ohio sites, we found clear separation in communities between all three soil fractions (Figure 4). Rhizoplane soils at the Ohio sites were also less diverse in bacteria than rhizosphere soils, indicating that only a small subset colonize that zone. The biologically active rhizosphere extends to about 4 mm from the root surface, with enzyme activity and oxygen concentration decreasing with increasing distance from the root (Kuzyakov & Razavi, 2019), likely creating gradients that drive microbial colonization at different spatial scales. Our inability to detect cultivation in the rhizosphere of the Michigan sites could result from the sampling method not being sensitive enough to pick up differences between soil fractions (i.e., not including rhizoplane samples). Although rhizosphere sampling is common, the methods employed often vary slightly, and small variations can cause difference in the microbes recovered (Barillot et al., 2013). Nonetheless, our inability to detect differences in the Michigan sites using standard methods is more likely a reflection of the strength of cultivation in the less dense stands.

We gleaned little evidence from putative functional descriptors of our microbial communities that suggests invasiveness of *Phragmites* is explained by differential cultivation of microbes in rhizosphere soils. In addition to the similarity in composition between native and non‐native lineages, both lineages harbored functionally similar microbial communities, consisting mostly of saprotrophic fungi, few known fungal or bacterial pathogens, and a small subset of mutualists (mainly AMF). AMF abundance in the rhizosphere also did not differ among sites. Non‐native *Phragmites* roots have been found to be more heavily colonized by fungi than native *Phragmites* with the differences being greatest in drier sites (Bickford et al., 2018). Therefore, while sites do not differ in AMF abundance in soils, recruitment into roots may differ between lineages. Still, given the low abundance of AMF in both studies, especially in wet sites in which the non‐native lineage is often highly successful, it is not likely a major driver of invasiveness in *Phragmites*. Lacking evidence to support the role of root‐associated microorganisms in fostering invasive properties in the non‐native *Phragmites* compared to the native, we suspect differences in plant performance arise due to other aspects of plant growth.

Although we saw no consistent evidence that *Phragmites* lineages cultivate different soil microbiomes, except for at high densities, it is possible that the response to soil microbes differs between lineages to a similar community of microbes. To investigate whether each lineage has a unique response to soil microbes, we would need to take an experimental approach and keep soil communities constant to see how the growth of each lineage is affected by soil microbes. In fact, experimental results indicate that native and non‐native lineages are capable of differential response to similar microbial communities (Bickford, 2020). It is also important to note that although both lineages seem to be cultivating compositionally similar communities, those microbes may negatively impact other native plants (Allen et al., 2018; Crocker et al., 2017), facilitating expansion after establishment.

Cumulatively, the results we report here and elsewhere provide little evidence to support the idea that non‐native *Phragmites* out‐performs native *Phragmites* by altering the composition and function of root‐associated microbial communities in soil. Alteration of the soil microbiome may occur in dense high‐density patches of native and non‐native *Phragmites*. However, those differences do not likely drive initial invasiveness and may in fact be consequences of alteration of the soil physical environment as non‐native *Phragmites* increases dominance and increases surrounding soil oxygen concentration relative to native *Phragmites*, so could potentially be important in later stages of maintaining invasion or expansion. Future research should experimentally examine the role that stand density and dominance play in differential microbial community cultivation, assess the belowground selective forces driving rhizosphere community composition, and evaluate their effects on range expansion and invasiveness.

## CONFLICT OF INTEREST

Authors have no conflict of interest to declare.

## AUTHOR CONTRIBUTIONS

**Wesley A. Bickford:** Conceptualization (lead); data curation (lead); formal analysis (lead); funding acquisition (supporting); investigation (lead); methodology (lead); project administration (equal); software (lead); validation (lead); visualization (lead); writing – original draft (lead); writing – review & editing (lead). **Donald R. Zak:** Conceptualization (equal); methodology (supporting); project administration (equal); supervision (equal); writing – original draft (supporting); writing – review & editing (supporting). **Kurt P. Kowalski:** Conceptualization (supporting); funding acquisition (lead); project administration (equal); supervision (equal); writing – original draft (supporting); writing – review & editing (supporting). **Deborah E. Goldberg:** Conceptualization (equal); project administration (equal); supervision (equal); writing – original draft (supporting); writing – review & editing (supporting).

### Open Research Badges

This article has earned an Open Data Badge for making publicly available the digitally‐shareable data necessary to reproduce the reported results. The data is available at https://doi.org/10.5066/P9HP8UXZ and https://doi.org/10.5066/P93BBZWU.

## Supporting information

Appendix S1

## Data Availability

DNA sequences: NCBI SRA Accession number PRJNA601975. Field and laboratory data: Peer‐reviewed data release on USGS ScienceBase (https://doi.org/10.5066/P9HP8UXZ). All code for bioinformatics and statistical analysis made public via peer‐reviewed USGS software release and deposited on code.usgs.gov (https://doi.org/10.5066/P93BBZWU).
