# Supplementary material for: Differences in rhizosphere microbial communities between native and non‐native Phragmites australis may depend on stand density
Source: Ecol Evol. 2020 Sep 29;10(20):11739–51. doi: 10.1002/ece3.6811 (PMC7593150; doi:10.1002/ece3.6811)
Supplement: Supplementary file 1 — Appendix S1 [file ECE3-10-11739-s001.docx]

Appendix S1

**Differences in Rhizosphere Microbial Communities Between Native and Non-Native *Phragmites australis* May Depend on Stand Density***.*

Running title: Rhizosphere microbes and plant invasions

**Authors:**

Wesley. A. Bickford^1,2*^, Donald R. Zak^2,3^, Kurt K. Kowalski^1,3^, and Deborah E. Goldberg^2^

^1^U.S. Geological Survey – Great Lakes Science Center, Ann Arbor, MI 48015, USA

^2^Department of Ecology and Evolutionary Biology, University of Michigan, Ann Arbor, MI 48109, USA

^3^School for Environment and Sustainability, University of Michigan, Ann Arbor, MI 48109, USA

*Author for correspondence: email – [wbickford@usgs.gov](mailto:wbickford@usgs.gov)

Manuscript accepted in *Ecology and Evolution*

**Table S1:** Site characteristics and environmental variables for a) all Michigan sites and b) Ohio transect sites. Michigan sites were chosen the same as those sampled in Bickford et al. (2018) except for those where patches no longer existed due to either herbicide management (Bullard Lake and Pointe Le Barb) or drowning by high lake water levels (Sturgeon Bay). The Bay city site was added to replace Bullard Lake as another lower Michigan site.

| **Site** | **Coordinates** | **Soil Map Unit** | **Coastal or Inland** | **No. Sampling locations** | **Saturation Levels**  **(# of each)** | **Soil C (%) mean (range)** | **Soil N (%) mean (range)** | **Soil P (mg/kg) mean (range)** | **Tissue C (%) mean (range)** | **Tissue N (%) mean (range)** | **Tissue P (%) mean (range)** |
| --- | --- | --- | --- | --- | --- | --- | --- | --- | --- | --- | --- |
| Bay City Rec Area (BC) | 43°40' 51”N | Belleville loamy sand | Coastal | 6 | Saturated (6) | 8.42 | 0.51 | 5.57 | 46.07 | 0.78 | 0.06 |
|  | 83°55' 7”W |  |  |  |  | (3.65 – 21.00) | (0.21 – 1.34) | (3.59 – 8.37) | (45.31 – 46.97) | (0.41 – 1.60) | (0.03 - 0.13) |
| Chelsea Farm (CH) | 42˚18’32”N | Houghton Muck | Inland | 5 | Unsaturated (2) | 15.28 | 0.78 | 4.47 | 44.91 | 1.14 | 0.07 |
|  | 84˚03’25”W |  |  |  | Saturated (3) | (8.24 - 32.24) | (0.32 – 1.99) | (0.30 – 7.00) | (44.55 – 45.19) | (0.63 – 1.58) | (0.04 – 0.15) |
| Cheboygan Marsh (CM) | 45˚39’27”N | Histosols and Aquents | Coastal | 6 | Saturated (1) | 7.06 | 0.35 | 3.15 | 45.63 | 0.76 | 0.06 |
|  | 84˚28’16”W |  |  |  | High Water (5) | (0.88 – 14.53) | (0 – 0.73) | (0 – 5.08) | (43.30 – 47.18) | (0.40 – 1.40) | (0.03 – 0.11) |
| Cecil Bay (CB) | 45˚44’52”N | Stony Lake Beach | Coastal | 6 | High Water (6) | 2.15 | 0.08 | 0.50 | 46.45 | 0.72 | 0.03 |
|  | 84˚50’57”W |  |  |  |  | (1.00 – 6.67) | (0 - 0.42) | (0 – 1.19) | (46.03 – 46.73) | (0.41 – 0.94) | (0.02 – 0.05) |
| Pointe aux Chenes (Rt2) | 45˚54’46”N | Leafriver mucky peat | Coastal | 6 | High Water (6) | 0.63 | 0.04 | 1.87 | 45.54 | 1.00 | 0.04 |
|  | 84˚52’20”W |  |  |  |  | (0.26 – 1.92) | (0.01 - 0.11) | (0.23 – 3.00) | (42.25 – 46.06) | (0.58 – 1.40) | (0.02 - 0.08) |
| Castle Rock (CR) | 45˚54’39”N | Eastport-Leafriver complex | Inland | 6 | Saturated (6) | 16.81 | 0.90 | 4.29 | 44.73 | 0.93 | 0.05 |
|  | 84˚44’18”W |  |  |  |  | (1.59 – 41.64) | (0.04 – 2.34) | (0.07 – 10.41) | (44.19 – 46.06) | (0.38 – 1.50) | (0.01 – 0.08) |

b)

| **Site** | **Coordinates** | **Soil Map Unit** | **Coastal or Inland** | **No. Sampling locations** | **Stand Types** | | | **Saturation Levels (No. of each)** | **Soil C (%) mean (range)** | **Soil N (%) mean (range)** | **Soil P (mg/kg) mean (range)** |
| --- | --- | --- | --- | --- | --- | --- | --- | --- | --- | --- | --- |
|  |  |  |  |  | **Bulk Soil Samples**  **(No. Native)**  **(No. Non-native)** | **Rhizosphere Samples**  **(No. Native)**  **(No. Non-native)** | **Rhizoplane Samples**  **(No. Native)**  **(No. Non-native)** |  |  |  |  |
| Ohio Transect 1 (T1) | 41°40' 33”N | Toledo silt loam | Coastal | 44 Total | 36 Mix / 8 Mono | 34 Mix / 8 Mono | 32 Mix / 6 Mono | Saturated (44) | 24.16 | 2.30 | 7.87 |
|  | 83°18'18"W |  |  | 21 Native  23 Non-native | (18 Mix / 3 Mono)  (18 Mix / 5 Mono) | (17 Mix / 3 Mono)  (17 Mix / 5 Mono) | (17 Mix / 1 Mono)  (15 Mix / 5 Mono) |  | (18.5 – 32.9) | (1.9 – 3.2) | (1.4 – 12.7) |
| Ohio Transect 2 (T2) | 41°40' 33"N | Toledo silt loam | Coastal | 25 Total | 20 Mix / 5 Mono | 19 Mix / 5 Mono | 16 Mix / 5 Mono | Saturated (25) | 17.29 | 1.41 | 12.26 |
|  | 83°17'33"W |  |  | 12 Native  13 Non-native | (10 Mix / 2 Mono)  (10 Mix / 3 Mono) | (10 Mix / 2 Mono)  (9 Mix / 3 Mono) | (8 Mix / 2 Mono)  (8 Mix / 3 Mono) |  | (8.5 – 39.3) | (0.7 – 2.6) | (1.0 – 40.5) |

**Table S2:** PCR Conditions and Primer Sequences

| **Primer Set** | **Primer** | **Primer Sequence** | **Fused Primer Length**^^[[1]](#footnote-1)^^ | **PCR Mastermix** | **PCR Conditions** | **Reference** |
| --- | --- | --- | --- | --- | --- | --- |
| Fungi | 5.8S_Fun | 5’-AACTTTYRRCAAYGGATCWCT-3’ | 65 bp | 5.0 uL 5x Buffer^^[[2]](#footnote-2)^*^  0.25 uL 20uM dNTPs  0.938 uL 20uM 5.8S  0.938 uL 20uM ITS4  0.25 uL Taq^*^  4 uL Template DNA^^[[3]](#footnote-3)^**^  13.625 uL H2O | Initial denaturation: 94 ̊C for 3 min, 27 cycles, denaturation: 94 ̊C for 30 s, annealing: 57 ̊C for 45 s, extension: 72 ̊C for 90 s (10 min final extension) | (Taylor et al. 2016) |
|  | ITS4_Fun | 5’-AGCCTCCGCTTATTGATATGCTTAART-3’ | 76 bp |  |  |  |
| Bacteria | 515F | 5’- GTGCCAGCMGCCGCGGTAA-3’ | 63 bp | 5.0 uL 5x Buffer^*^  0.5 uL 20uM dNTPs  0.5 uL 20uM 515F  0.5 uL 20uM 806R  0.5 uL Taq^*^  2 uL Template DNA^**^  18 uL H2O | Initial denaturation: 95 ̊C for 2 min, 30 cycles, denaturation: 95 ̊C for 20 s, annealing: 55 ̊C for 15 s, extension: 72 ̊C for 5 min (10 min final extension) | (Kozich et al. 2013) |
|  | 806R | 5’- GGACTACHVGGGTWTCTAAT’3’ | 69 bp |  |  |  |
| Oomycetes  (First round) | 5.8 SR | 5’-TCGATGAAGAACGCAGCG -3’ | - | 2.5 uL 10x Buffer^*^  0.5 uL BSA  0.5 uL 20uM dNTPs  0.625 uL 20uM 5.8 SR  0.625 uL 20uM LR7  0.5 uL Taq^*^  2 uL Template DNA^**^  17.75 uL H2O | Initial denaturation: 94 ̊C for 5 min, 30 cycles, denaturation: 94 ̊C for 30 s, annealing: 47 ̊C for 90 s, extension: 72 ̊C for 60 s (10 min final extension) | (Vilgalys and Hester 1990) |
|  | LR7 | 5’-TACTACCACCAAGATCT-3’ | - |  |  |  |
| Oomycetes  (Second Round) | ITS3oo | 5’-AGTATGYYTGTATCAGTGTC-3’ | 64 bp | 5.0 uL 5x Buffer^*^  0.25 uL 20uM dNTPs  0.125 uL 20uM Oom1f  0.125 uL 20uM Oom1r  0.75 uL DMSO  0.25 uL Taq^*^  1 uL Template DNA^**^  17.50 uL H2O | Initial denaturation: 95 ̊C for 15 min, 30 cycles, denaturation: 95 ̊C for 30 s, annealing: 55 ̊C for 30 s, extension: 72 ̊C for 60 s (10 min final extension) | (Riit et al. 2016) |
|  | ITS4 | 5’-TCCTCCGCTTATTGATATGC-3’ | 69 bp |  |  |  |

a) Bacteria


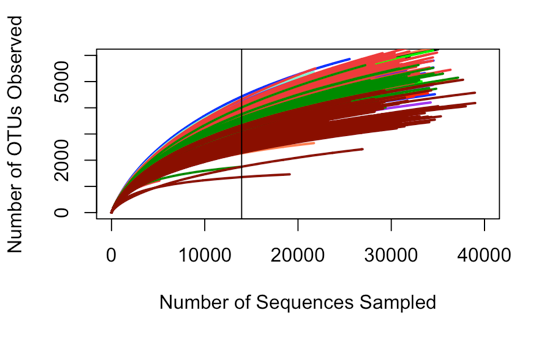


b) Fungi


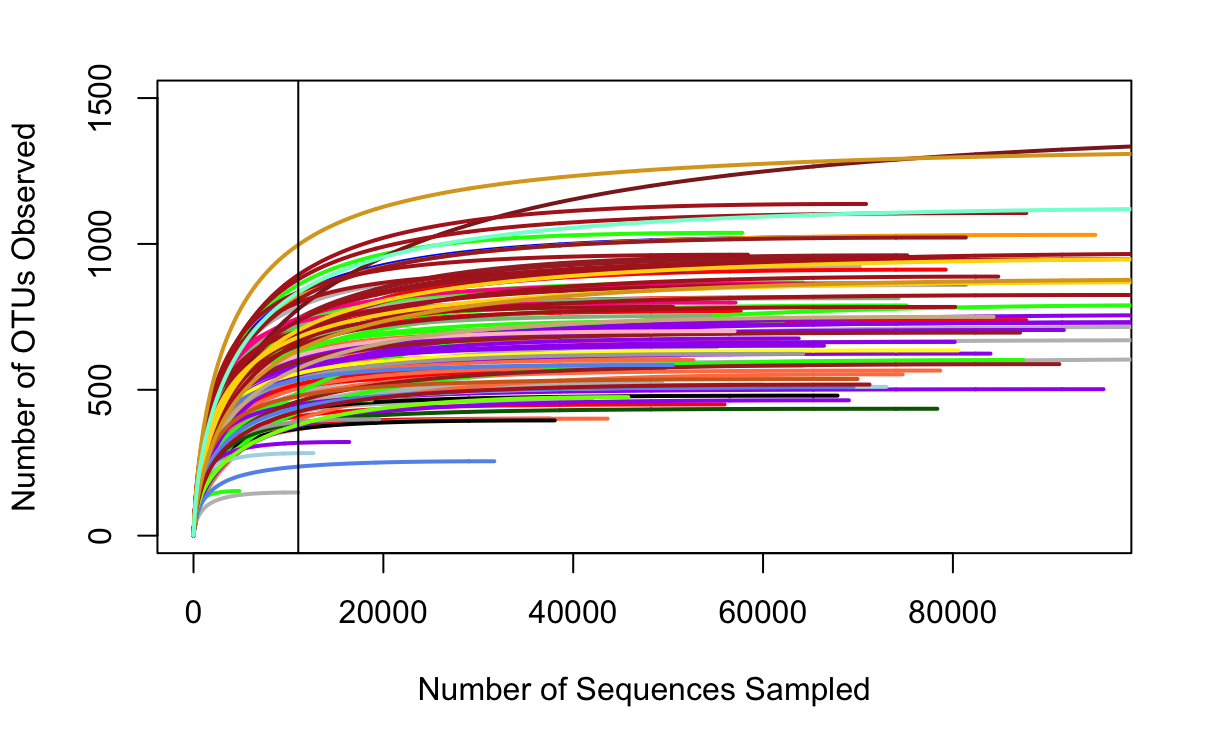


c) Oomycetes


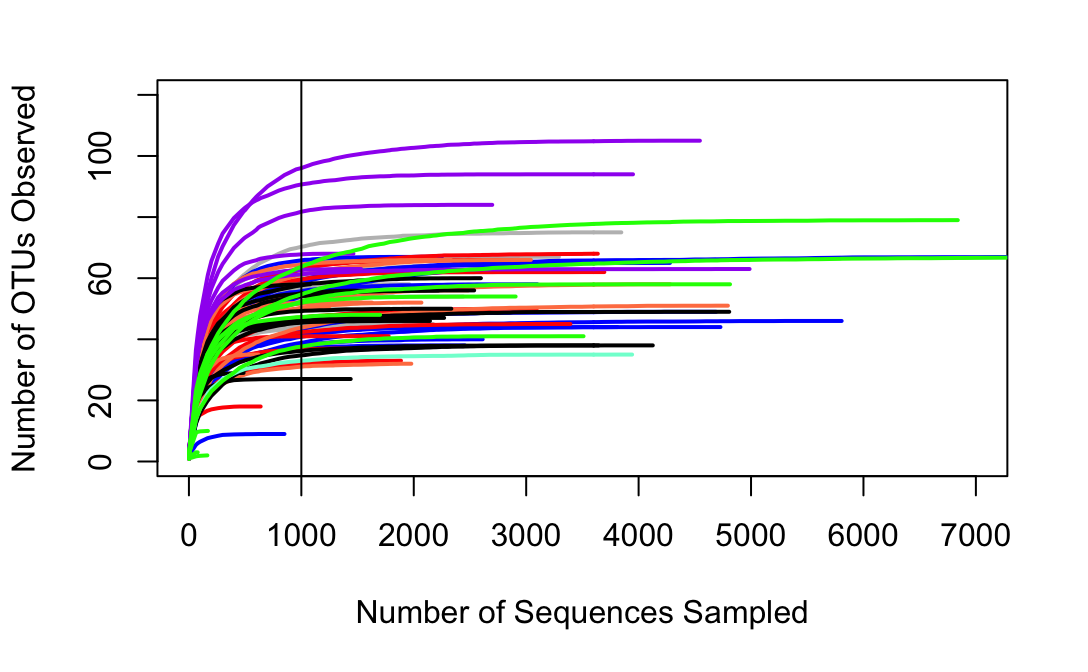


**Fig. S1:** Rarefaction curves for a) bacteria b) fungi and c) oomycete sequences. Colors indicate sampling locations. Vertical lines indicate subsample sequence amount.

**Table S3:** a) ANCOVA table showing the relationship between site, lineage, soil saturation, and nutrients on relative abundance of bacterial phyla. b) ANOVA table showing the relationship between site and plant lineage on the relative abundance of bacterial phyla.

|  | **r^2^** | | | | **Lineage** | | **Saturation** | | **SoilN** | | **SoilP** | | **Sat:Lin** | |
| --- | --- | --- | --- | --- | --- | --- | --- | --- | --- | --- | --- | --- | --- | --- |
|  | **Residual df** | **Multiple r^2^** | **Adjusted r^2^** | ***P*** | **F** | ***P*** | **F** | ***P*** | **F** | ***P*** | **F** | ***P*** | **F** | ***P*** |
| Proteobacteria RA | 26 | **0.560** | **0.458** | **<0.001** | 1.68 | 0.206 | **14.99** | **<0.001** | 0.01 | 0.939 | 3.17 | 0.086 | 1.122 | 0.280 |
| Bacteriodetes RA | 26 | 0.087 | 0.000 | 0.865 | 0.14 | 0.708 | 0.32 | 0.575 | 0.10 | 0.757 | 0.12 | 0.732 | 0.38 | 0.542 |
| Acidobacteria RA | 26 | **0.756** | **0.670** | **<0.001** | 1.12 | 0.299 | **62.15** | **<0.001** | 0.42 | 0.523 | **6.05** | **0.021** | 0.02 | 0.882 |
| Chloroflexi RA | 26 | 0.355 | 0.206 | 0.057 | 0.67 | 0.420 | **12.38** | **0.002** | 0.53 | 0.472 | 0.42 | 0.524 | 0.14 | 0.710 |
| Planctomycetes RA | 26 | 0.168 | 0.000 | 0.528 | 1.53 | 0.227 | 0.02 | 0.896 | 0.116 | 0.737 | 1.18 | 0.288 | 0.00 | 0.985 |
| Verucomicrobia RA | 26 | 0.271 | 0.102 | 0.185 | 2.25 | 0.146 | **7.29** | **0.012** | 0.09 | 0.758 | 0.00 | 0.954 | 0.00 | 0.978 |

|  | **Lineage** | | | **Site** | | | **Site X Lineage** | | |
| --- | --- | --- | --- | --- | --- | --- | --- | --- | --- |
|  | **df** | **F** | **P** | **df** | **F** | **P** | **df** | **F** | **P** |
| Proteobacteria RA | 1 | 2.98 | 0.098 | **5** | **6.67** | **<0.001** | 5 | 1.01 | 0.435 |
| Bacteriodetes RA | 1 | 0.41 | 0.528 | **5** | **9.51** | **<0.001** | **5** | **4.36** | **0.006** |
| Acidobacteria RA | 1 | 4.17 | 0.053 | **5** | **17.81** | **<0.001** | 5 | 1.64 | 0.189 |
| Chloroflexi RA | 1 | 1.29 | 0.268 | 5 | 1.38 | 0.268 | 5 | 1.14 | 0.368 |
| Planctomycetes RA | 1 | 1.56 | 0.224 | 5 | 2.51 | 0.059 | 5 | 1.51 | 0.224 |
| Verucomicrobia RA | 1 | 4.21 | 0.052 | 5 | 1.46 | 0.240 | 5 | 2.05 | 0.109 |

**Fig S2:** Regression of relative abundance of bacterial phyla vs. soil saturation. Trend lines indicate significant correlation (P < 0.05) between saturation and the given bacterial phylum.


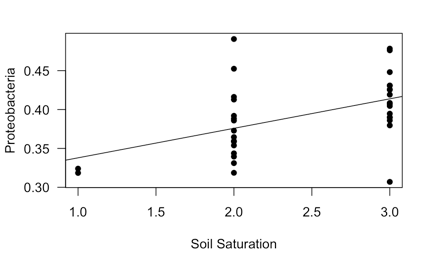

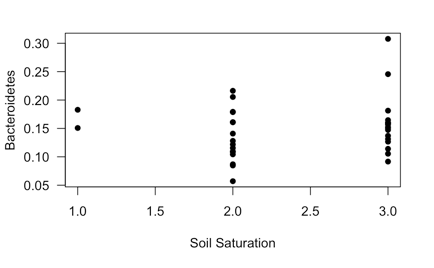

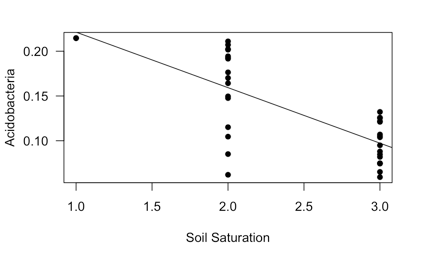

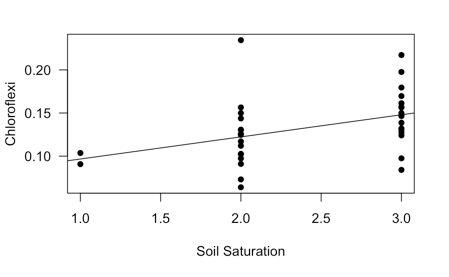

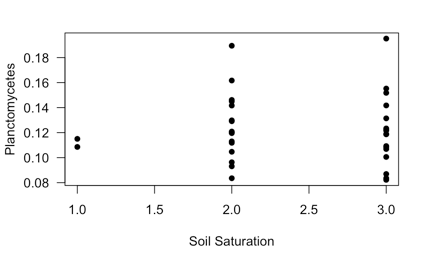

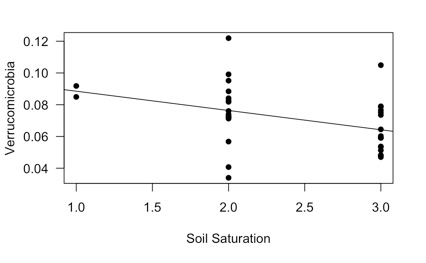


**Table S4:** a) ANCOVA table showing the relationship between site, lineage, soil saturation, and nutrients on relative abundance of fungal phyla. b) ANOVA table showing the relationship between site and plant lineage on the relative abundance of fungal phyla.

|  | r^2^ | | | | Lineage | | Saturation | | SoilN | | SoilP | | Sat:Lin | |
| --- | --- | --- | --- | --- | --- | --- | --- | --- | --- | --- | --- | --- | --- | --- |
|  | Residual df | Multiple r^2^ | Adjusted r^2^ | *P* | F | *P* | F | *P* | F | *P* | F | *P* | F | *P* |
| Unclassified RA | 25 | 0.172 | 0.000 | 0.532 | 0.37 | 0.547 | 3.54 | 0.071 | 0.18 | 0.679 | 0.13 | 0.723 | 0.46 | 0.504 |
| Ascomycota RA | 25 | 0.160 | 0.000 | 0.583 | 0.73 | 0.402 | 2.08 | 0.162 | 0.69 | 0.415 | 0.89 | 0.353 | 0.01 | 0.925 |
| Basidiomycota RA | 25 | 0.189 | 0.000 | 0.465 | 1.60 | 0.218 | 0.24 | 0.630 | 0.87 | 0.359 | 0.89 | 0.355 | 1.90 | 0.181 |
| Rozellomycota RA | 25 | 0.299 | 0.000 | 0.932 | 0.33 | 0.568 | 0.43 | 0.517 | 0.34 | 0.567 | 0.23 | 0.638 | 0.04 | 0.841 |
| Mortierellomycota RA | 25 | **0.535** | **0.424** | **0.002** | 0.21 | 0.652 | **24.67** | **<0.001** | 0.47 | 0.501 | 0.36 | 0.552 | 3.06 | 0.093 |
| Glomeromycota RA | 25 | 0.289 | 0.118 | 0.165 | 3.40 | 0.077 | 4.15 | 0.052 | 0.30 | 0.587 | 0.90 | 0.351 | 1.11 | 0.302 |

|  | Lineage | | | Site | | | Site X Lineage | | |
| --- | --- | --- | --- | --- | --- | --- | --- | --- | --- |
|  | df | F | P | df | F | P | df | F | P |
| Unclassified RA | 1 | 0.18 | 0.679 | 5 | 2.65 | 0.051 | 5 | 0.59 | 0.709 |
| Ascomycota RA | 1 | 0.46 | 0.506 | 5 | 1.97 | 0.123 | 5 | 0.56 | 0.730 |
| Basidiomycota RA | 1 | 1.29 | 0.268 | 5 | 1.56 | 0.211 | 5 | 0.57 | 0.722 |
| Rozellomycota RA | 1 | 0.30 | 0.590 | 5 | **3.99** | **0.010** | 5 | 0.23 | 0.947 |
| Mortierellomycota RA | 1 | 1.64 | 0.213 | 5 | **8.88** | **<0.001** | **5** | **2.83** | **0.040** |
| Glomeromycota RA | 1 | **5.03** | **0.035** | 5 | 1.52 | 0.225 | 5 | 2.14 | 0.099 |


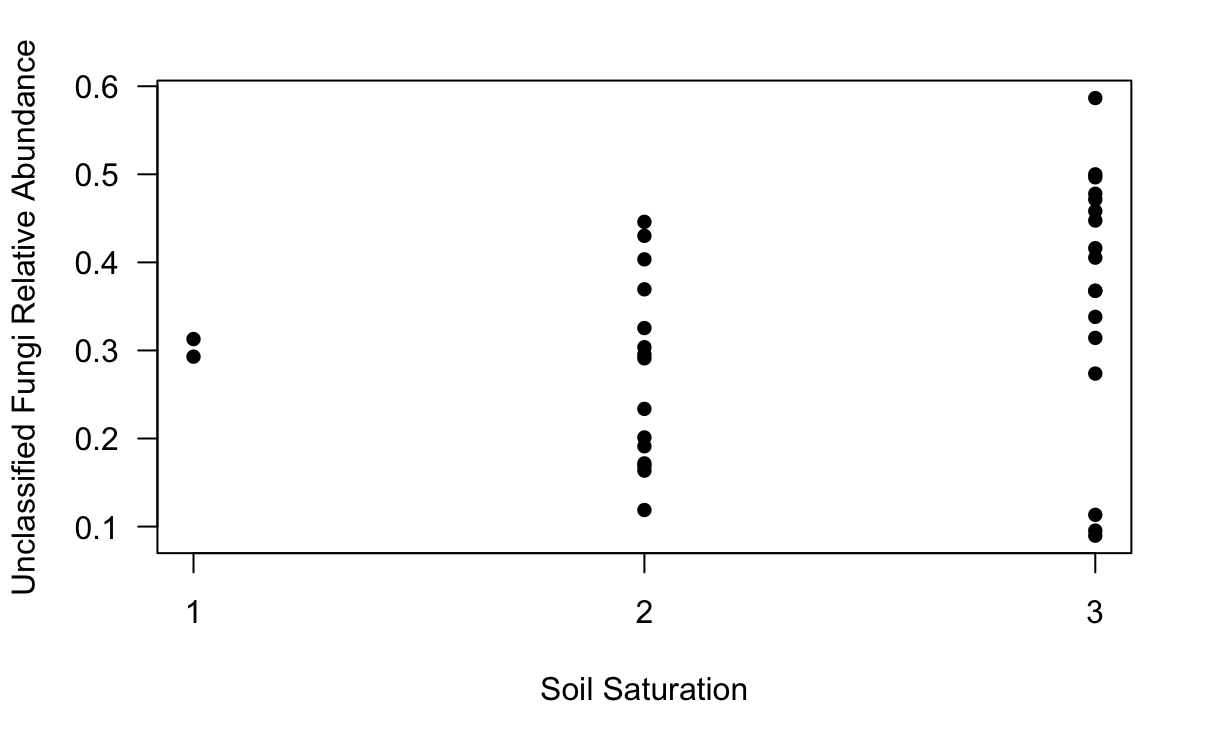

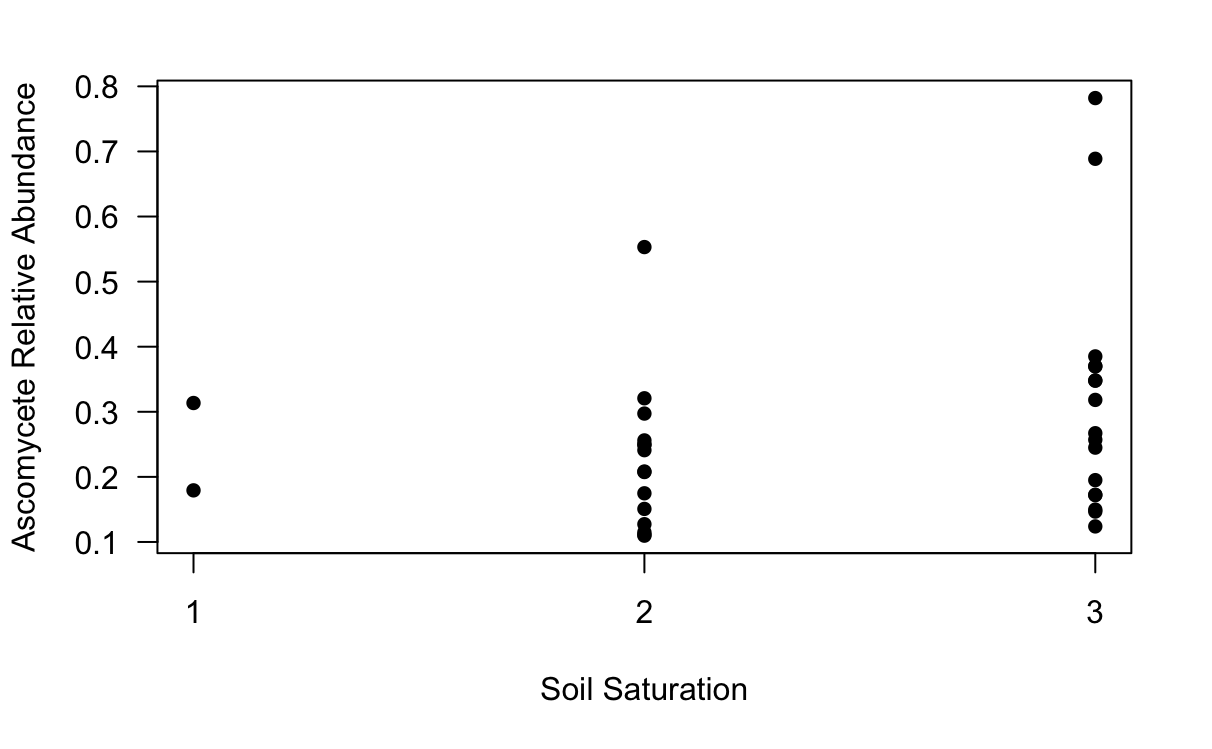

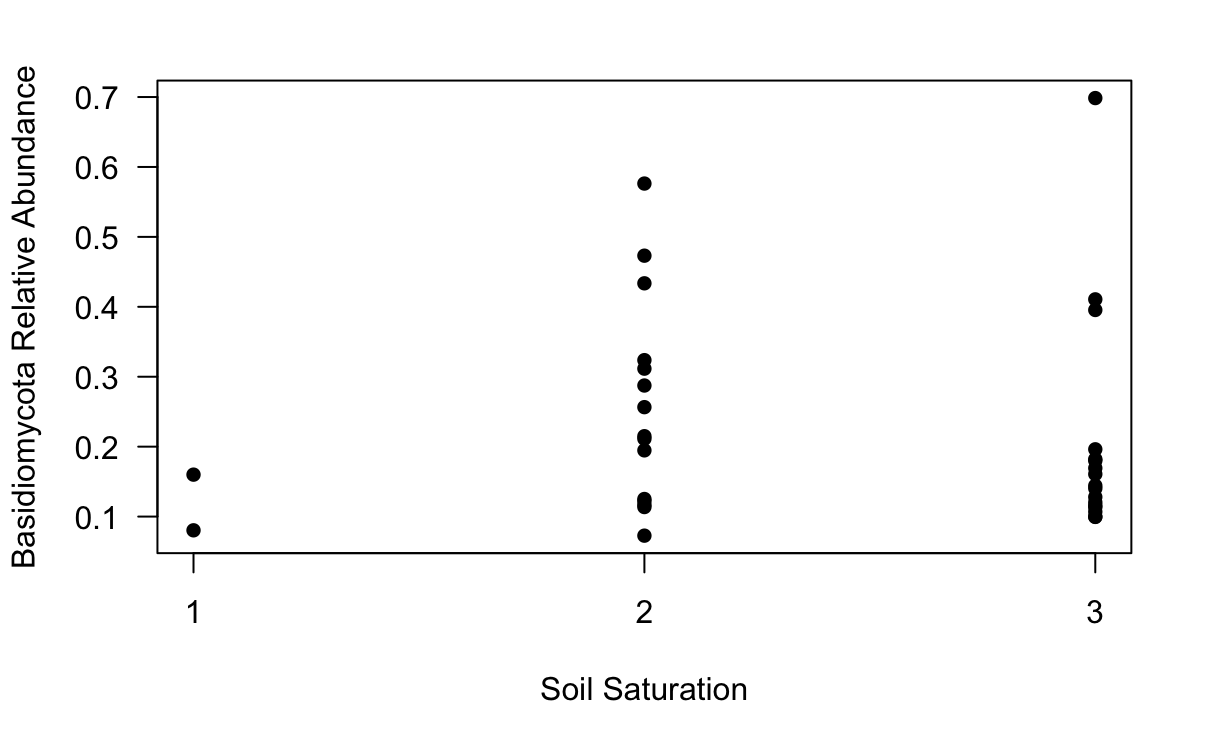


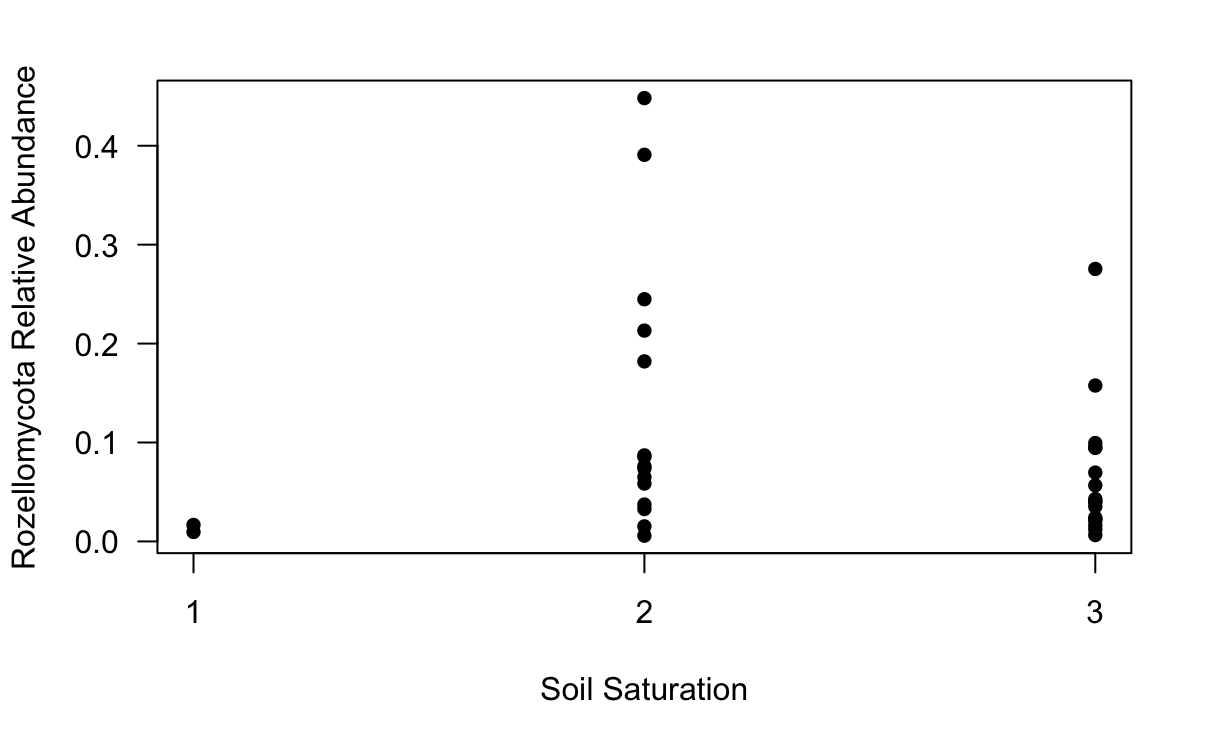

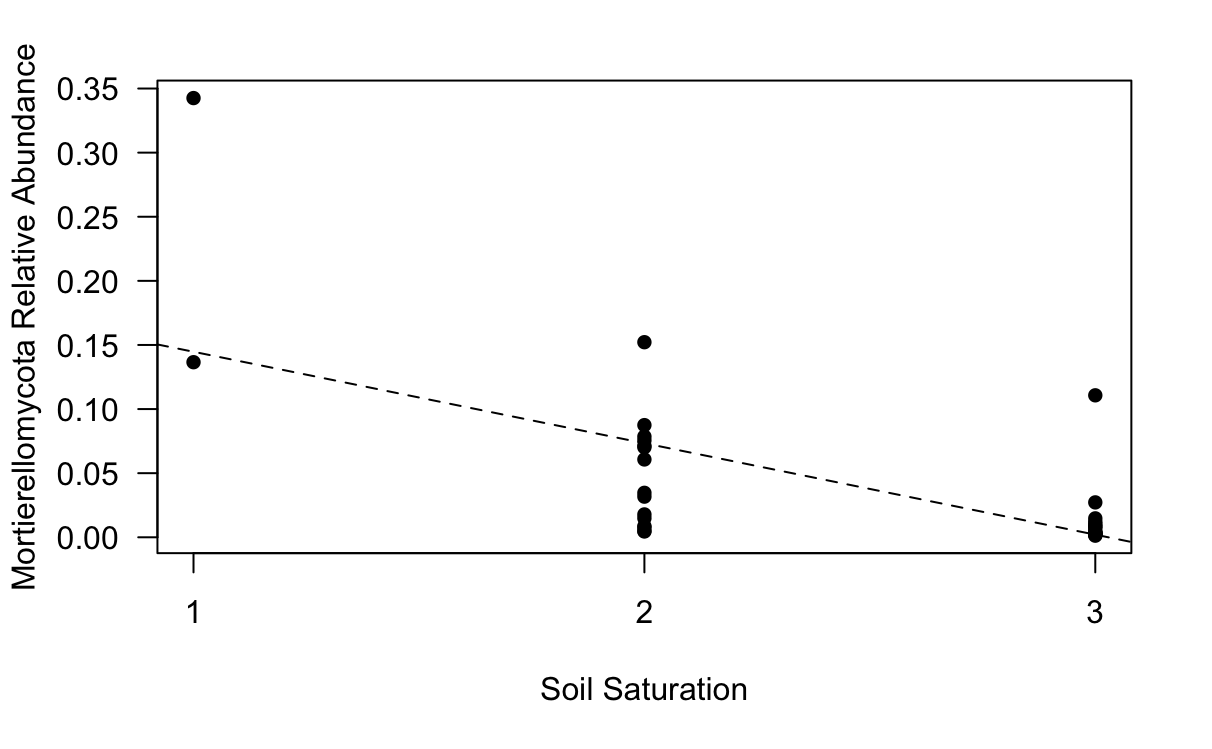

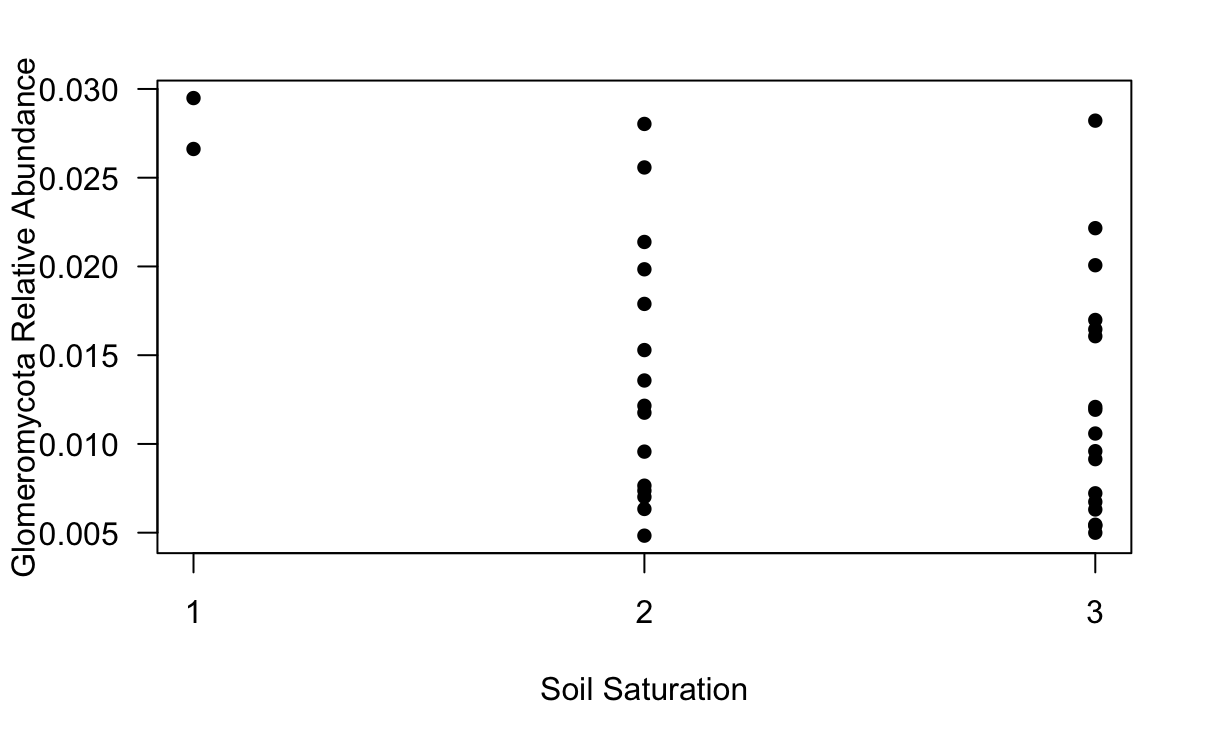


**Fig S3:** Regression of relative abundance of fungal phyla vs. soil saturation. Trend lines indicate significant correlation (P < 0.05) between saturation and the given fungal phylum.

**Table S5:** a) ANCOVA table showing the relationship between site, lineage, soil saturation, and nutrients on relative abundance of oomycete families. b) ANOVA table showing the relationship between site and plant lineage on the relative abundance of oomycete families.

| a) | **r^2^** | | | | **Lineage** | | **Saturation** | | **SoilN** | | **SoilP** | | **Sat:Lin** | |
| --- | --- | --- | --- | --- | --- | --- | --- | --- | --- | --- | --- | --- | --- | --- |
|  | **Residual df** | **Multiple r^2^** | **Adjusted r^2^** | ***P*** | **F** | ***P*** | **F** | ***P*** | **F** | ***P*** | **F** | ***P*** | **F** | ***P*** |
| Pythiaceae RA | 21 | **0.463** | **0.309** | **0.028** | **5.40** | **0.030** | 3.57 | 0.073 | 0.53 | 0.473 | 0.59 | 0.453 | **7.11** | **0.014** |
| Unclassified RA | 21 | **0.482** | **0.334** | **0.020** | **5.33** | **0.031** | 4.16 | 0.054 | 0.79 | 0.383 | 0.83 | 0.374 | **7.09** | **0.015** |
| Saprolegniaceae RA | 21 | 0.120 | 0.000 | 0.817 | 0.51 | 0.485 | 0.10 | 0.758 | 0.47 | 0.500 | 0.35 | 0.559 | 0.57 | 0.460 |
| Leptoleniaceae RA | 21 | 0.176 | 0.000 | 0.619 | 1.09 | 0.309 | 0.37 | 0.552 | 0.03 | 0.864 | 0.39 | 0.540 | 0.08 | 0.778 |

b)

|  | **Lineage** | | | **Site** | | | **Site X Lineage** | | |
| --- | --- | --- | --- | --- | --- | --- | --- | --- | --- |
|  | **df** | **F** | **P** | **df** | **F** | **P** | **df** | **F** | **P** |
| Pythiaceae RA | 1 | 3.93 | 0.064 | **5** | **3.51** | **0.023** | **5** | **3.60** | **0.021** |
| Unclassified RA | 1 | 3.64 | 0.074 | **5** | **3.68** | **0.019** | **5** | **3.76** | **0.018** |
| Saprolegniaceae RA | 1 | 0.47 | 0.501 | 5 | 0.54 | 0.741 | 5 | 0.69 | 0.636 |
| Leptoleniaceae RA | 1 | 0.49 | 0.495 | 5 | 0.57 | 0.726 | 5 | 0.57 | 0.726 |


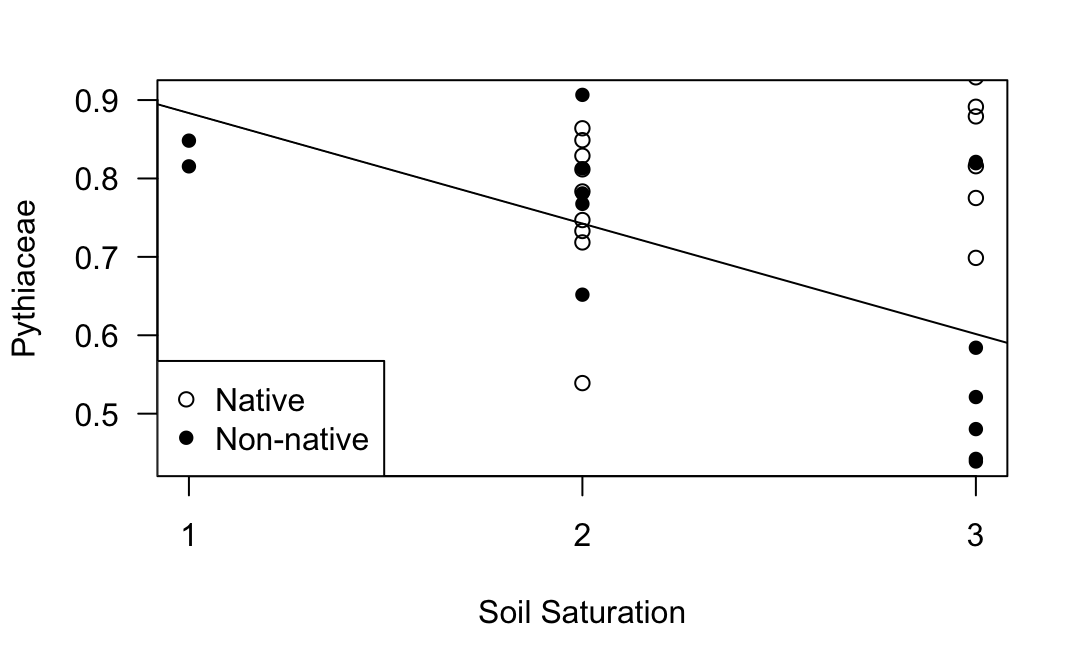

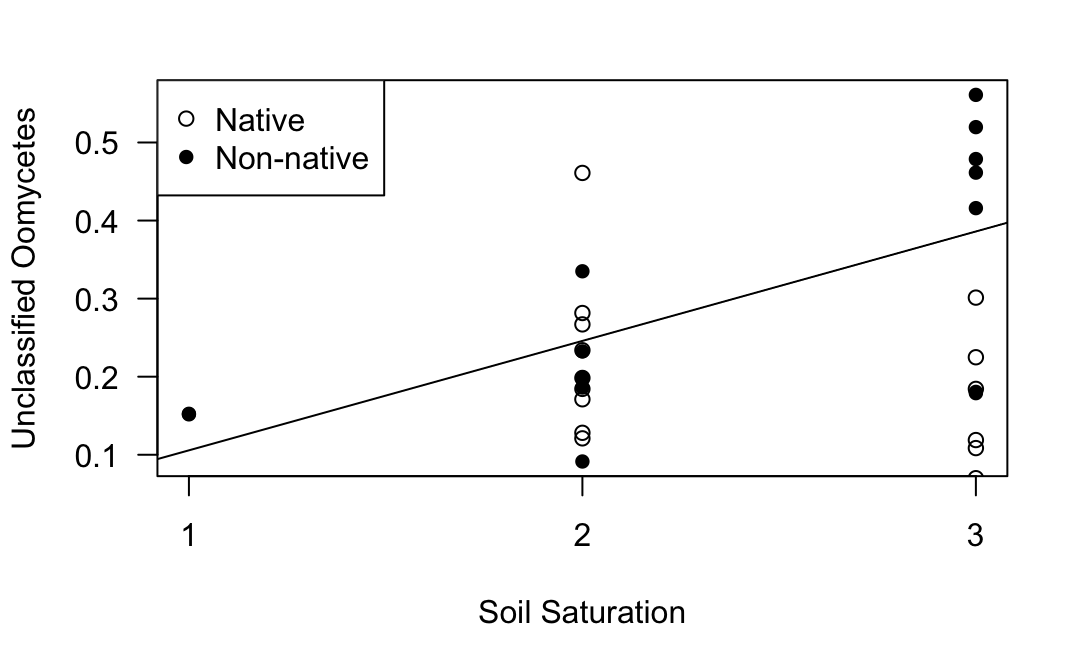


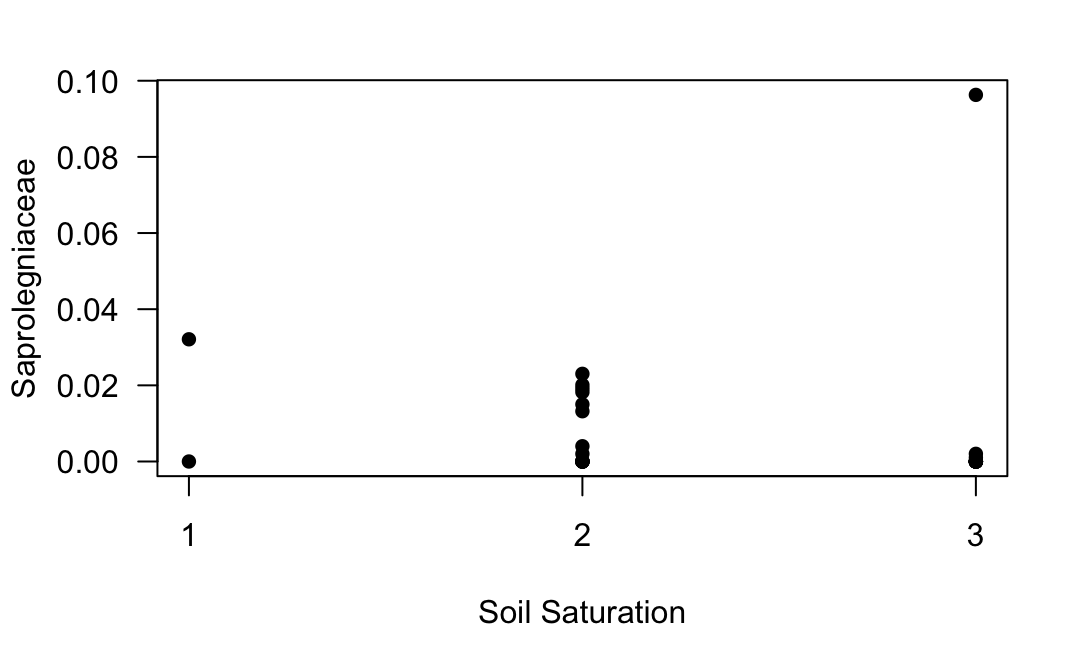

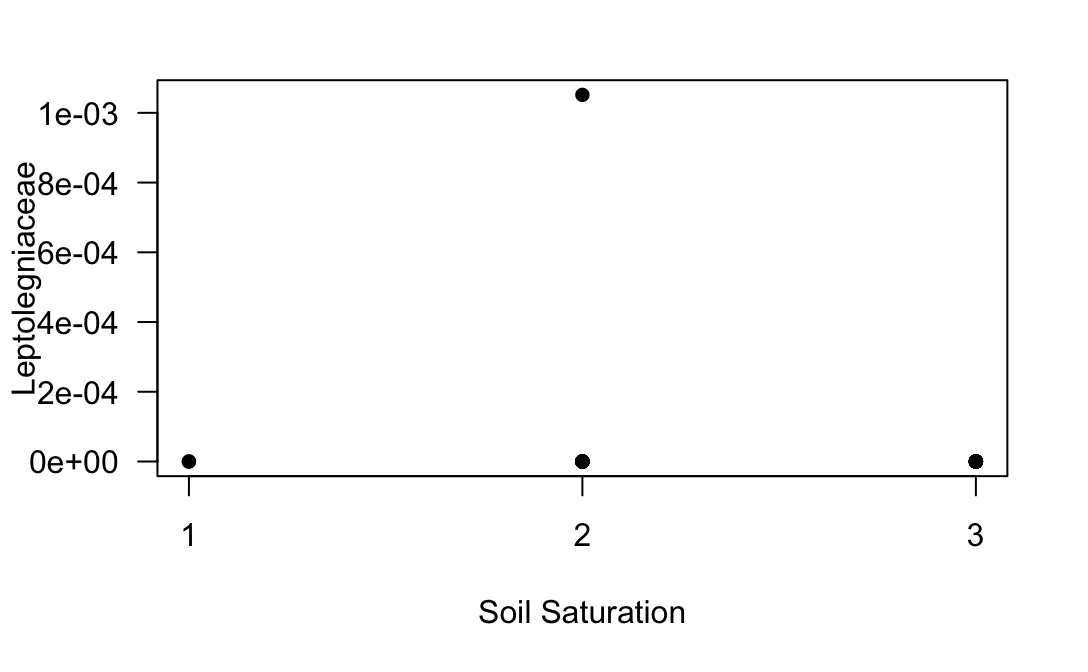


**Fig S4** Regression of relative abundance of oomycete families vs. soil saturation. Soil saturation levels indicate degree of saturation: 1=Unsaturated, 2=Saturated, 3=Standing water. In a) and b) trendlines indicate significant correlations (*P* < 0.05) between saturation and relative abundance in non-native plants only. Native correlations non-significant (see Table S3.4)


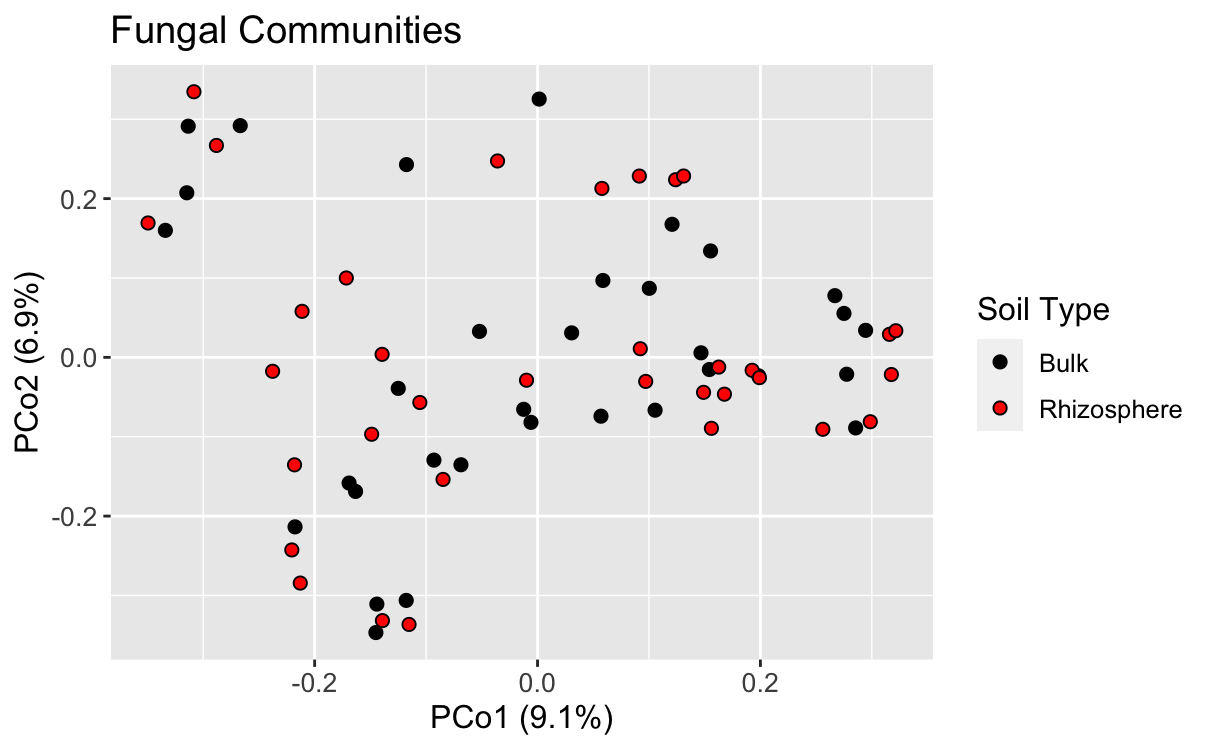

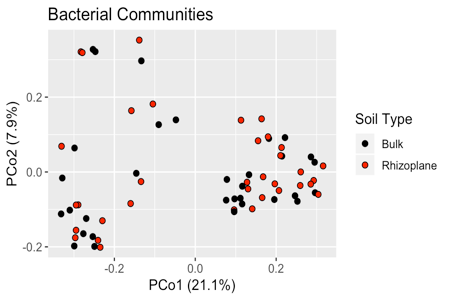

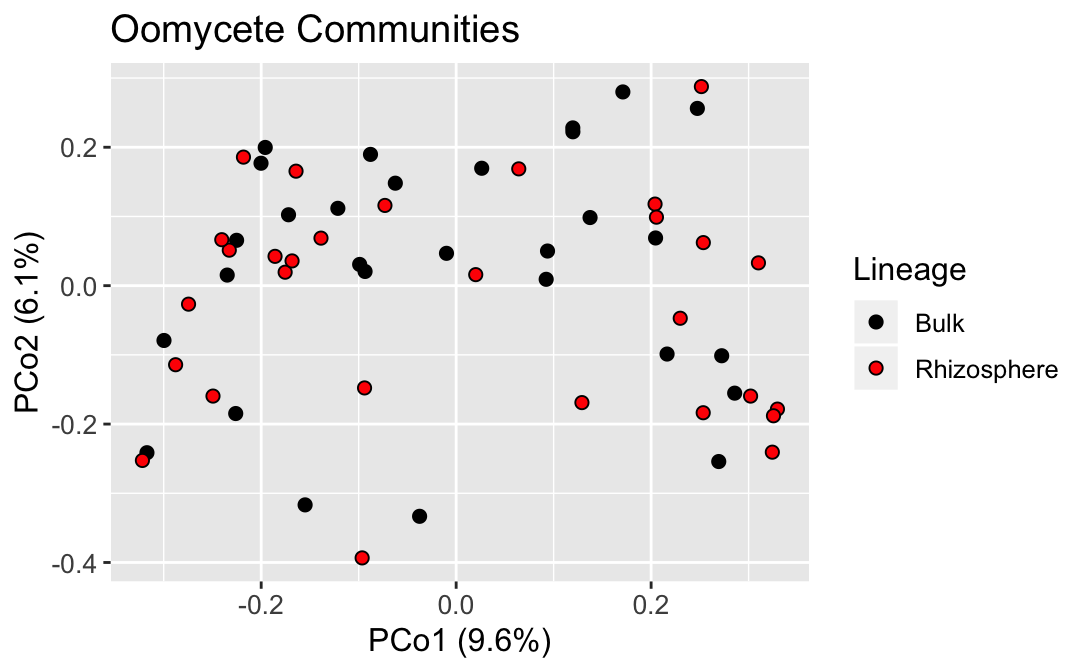


**a)**

**b)**

**c)**

**Fig. S5:** Principle coordinate analysis of Bray-Curtis distances between bulk and rhizosphere **a)** bacterial, **b)** fungal, and **c)** oomycete communities. Bacterial (Per-MANOVA P = 0.969; PermDISP P = 0.958), fungal (Per-MANOVA P = 0.979; PermDISP P = 0.511), and oomycete (Per-MANOVA P = 0.86; PermDISP P = 0.545) communities did not differ between bulk and rhizosphere soils.


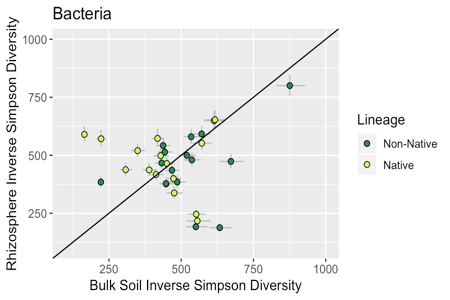

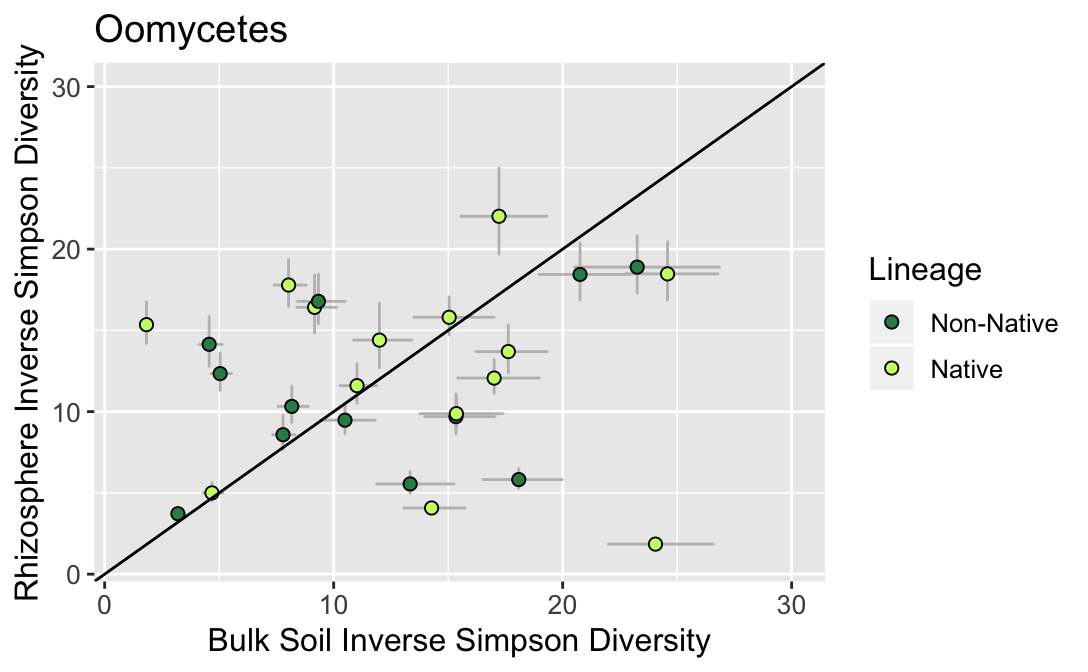

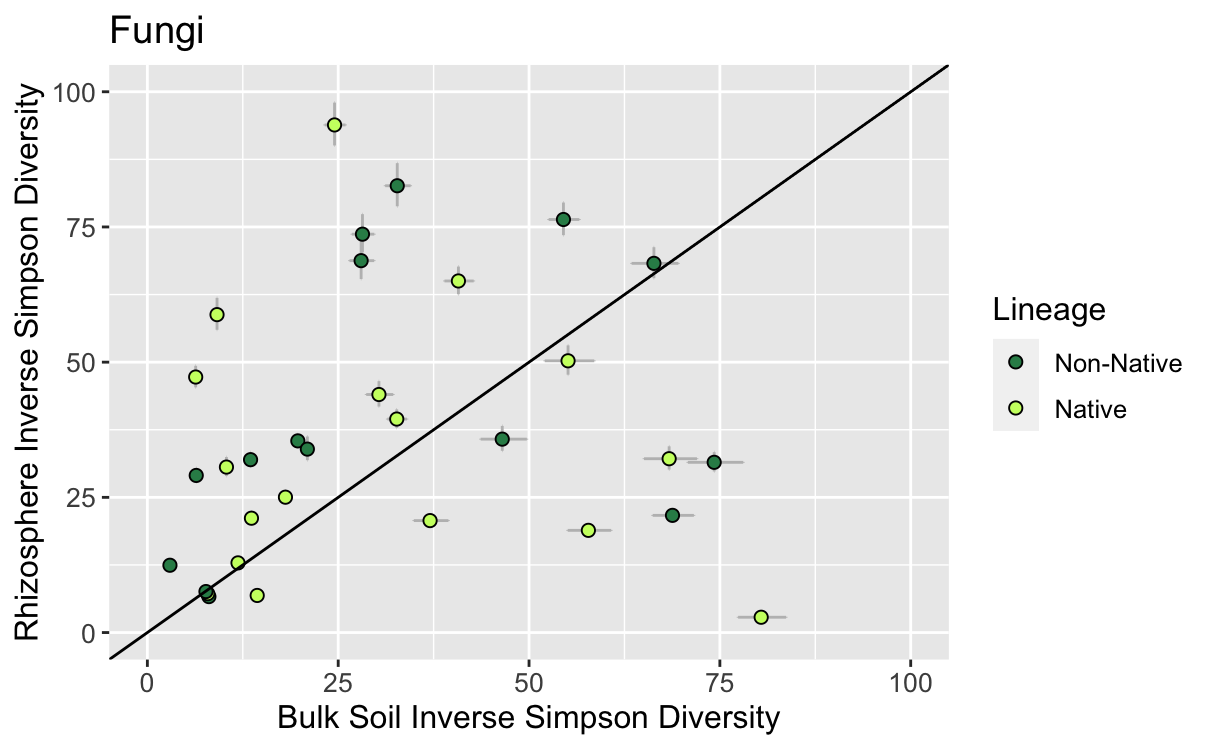


**Fig.S6:** Inverse Simpson diversity of **a)** bacteria, **b)** fungi, and **c)** oomycetes in paired rhizosphere and bulk soils. Black line indicates 1:1 relationship between bulk and rhizosphere diversity.


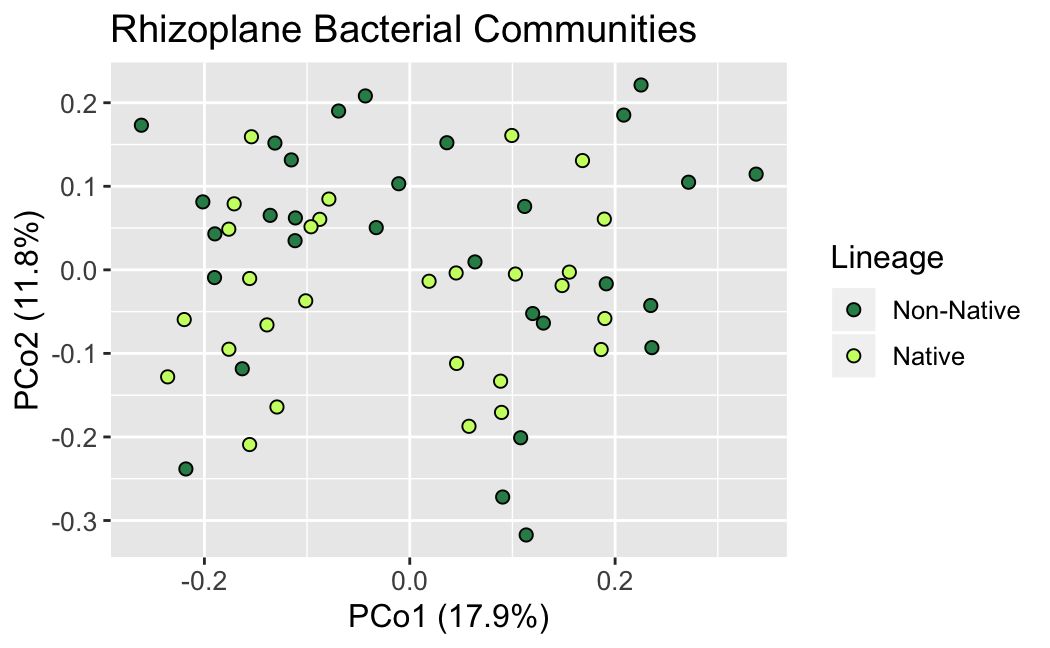


b)

a)


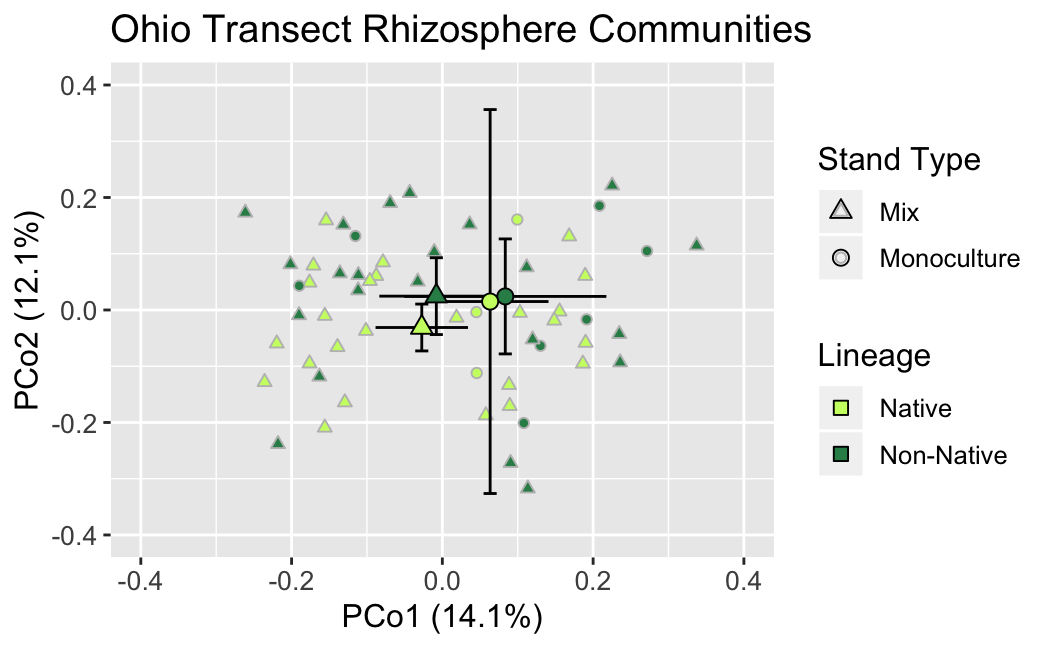


Ohio Transect Rhizoplane Communities

**Fig. S7:** a) Rhizoplane communities by lineage PCoA b) Rhizoplane community differences by stand type (mix and monoculture) and plant lineage (native and non-native)

**Table S6:** Pair-wise Per-MANOVA comparing **rhizosphere** bacterial communities in various zones of *Phragmites*. InvMix: Non-native *Phragmites* focal plant in a mixed zone, NatMix: Native focal plant in a mixed zone, InvMono: Non-native focal plant in a monoculture zone, NatMono: Native focal plant in a monoculture zone. P-values adjusted using a Bonferroni correction.

| **Pairs** | **F-value** | **r^2^** | ***P*-value** | ***P*-adjusted*** |
| --- | --- | --- | --- | --- |
| InvMix vs NatMix | 0.89 | 0.017 | 0.601 | 1.000 |
| InvMix vs InvMono | 1.05 | 0.032 | 0.348 | 1.000 |
| InvMix vs NatMono | 1.67 | 0.054 | 0.042 | 0.252 |
| NatMix vs InvMono | 1.21 | 0.036 | 0.188 | 1.000 |
| NatMix vs NatMono | 1.30 | 0.043 | 0.125 | 0.750 |
| InvMono vs NatMono | 2.08 | 0.159 | 0.008 | 0.048 |

**Table S7:** Pair-wise Per-MANOVA comparing **rhizoplane** bacterial communities in various zones of *Phragmites*. InvMix: Non-native *Phragmites* focal plant in a mixed zone, NatMix: Native focal plant in a mixed zone, InvMono: Non-native focal plant in a monoculture zone, NatMono: Native focal plant in a monoculture zone. *P-values adjusted using a Bonferroni correction.

| **Pairs** | **F-value** | **r^2^** | ***P*-value** | ***P*-adjusted*** |
| --- | --- | --- | --- | --- |
| InvMix vs NatMix | 1.05 | 0.023 | 0.324 | 1.000 |
| InvMix vs InvMono | 0.96 | 0.033 | 0.442 | 1.000 |
| InvMix vs NatMono | 0.95 | 0.040 | 0.451 | 1.000 |
| NatMix vs InvMono | 1.60 | 0.049 | 0.042 | 0.252 |
| NatMix vs NatMono | 1.08 | 0.040 | 0.322 | 1.000 |
| InvMono vs NatMono | 0.91 | 0.092 | 0.600 | 1.000 |


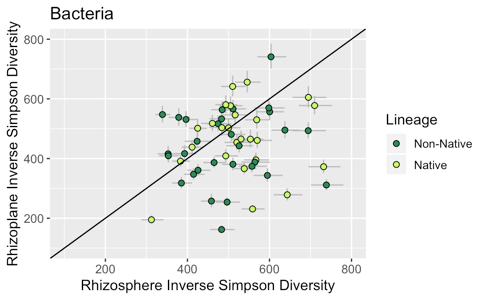

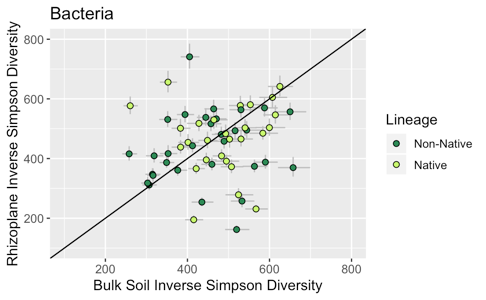


**Fig. S8:** Inverse Simpson diversity in paired **a)** rhizoplane and bulk soils and **b)** rhizoplane and rhizosphere soils from the Ohio transect sites. Black line indicates 1:1 relationship between bulk and rhizosphere diversity. Overall, rhizosphere soils were more diverse then the adjacent paired bulk soil (t = -2.799, df = 64, P = 0.007) and more diverse than paired rhizoplane soils (t = -3.059, df = 54, P = 0.003). Rhizoplane soils were not different in diversity from bulk soil (t = 0.786, df = 57, P = 0.435).

1. Full fused primer included Illumina adapter (29-bp forward; 24-bp reverse), 8-bp unique barcode, 10-bp pad, 2-bp linker followed by the gene specific primer). Reverse adapter was used with forward primer, Forward adapter with reverse. [↑](#footnote-ref-1)
2. * Phusion High Fidelity PCR Kit (New England BioLabs) [↑](#footnote-ref-2)
3. ** DNA was diluted to 5-10 ng per reaction [↑](#footnote-ref-3)
